# Metabolomics and Lipidomics Studies in Pediatric Type 1 Diabetes: Biomarker Discovery for the Early Diagnosis and Prognosis

**DOI:** 10.1155/2023/6003102

**Published:** 2023-07-13

**Authors:** Yaru Liu, Guanping Dong, Ke Huang, Ye Hong, Xuefeng Chen, Mingqiang Zhu, Xiaoqiang Hao, Yan Ni, Junfen Fu

**Affiliations:** Department of Endocrinology, Children's Hospital, Zhejiang University School of Medicine, National Clinical Research Center for Child Health, National Children's Regional Medical Center, Hangzhou 310052, China

## Abstract

**Aim:**

Type 1 diabetes (T1D) is an autoimmune disease with heterogeneous risk factors. Metabolic perturbations in the pathogenesis of the disease are remarkable to illuminate the interaction between genetic and environmental factors and how islet immunity and overt diabetes develop. This review aimed to integrate the metabolic changes of T1D to identify potential biomarkers for predicting disease progression based on recent metabolomics and lipidomics studies with parallel methodologies.

**Methods:**

A total of 18 metabolomics and lipidomics studies of childhood T1D during the last 15 years were reviewed. The metabolic fingerprints consisting of 41 lipids and/or metabolite classes of subjects with islet autoantibodies, progressors of T1D, and T1D children were mapped in four-time dimensions based on a tentative effect-score rule.

**Results:**

From birth, high-risk T1D subjects had decreased unsaturated triacylglycerols, unsaturated phosphatidylcholines (PCs), sphingomyelins (SMs), amino acids, and metabolites in the tricarboxylic acid (TCA) cycle. On the contrary, lysophosphatidylcholines (LPCs) and monosaccharides increased. And LPCs and branched-chain amino acids (BCAAs) were elevated before the appearance of islet autoantibodies but were lowered after seroconversion. Choline-related lipids (including PCs, SMs, and LPCs), BCAAs, and metabolites involved in the TCA cycle were identified as consensus biomarkers potentially predicting the development of islet autoimmunity and T1D. Decreased LPCs and amino acids indicated poor glycemic control of T1D, while elevated lysophosphatidylethanolamines and saturated PCs implied good glycemic control. Further pathway analysis revealed that biosynthesis of aminoacyl-tRNA, BCAAs, and alanine, aspartate, and glutamate metabolism were significantly enriched. Moreover, established cohort studies and predictive statistical models of pediatric T1D were also summarized.

**Conclusion:**

The metabolic profile of high-risk T1D subjects and patients demonstrated significant changes compared with healthy controls. This integrated analysis provides a comprehensive overview of metabolic features and potential biomarkers in the pathogenesis and progression of T1D.

## 1. Introduction

Type 1 diabetes (T1D) is a chronic immune-mediated disease characterized by dysglycemia and lifelong insulin dependence. T1D develops primarily in children and young adults, spanning a wide range of ages at the onset of symptoms [1, 2]. The incidence of T1D varies from the highest level of up to 60 cases to relatively low levels of 0.1–2.2 cases per 1,00,000 children, with an average annual increase of 3%-4% during the past three decades [2, 3]. Although the etiology of T1D requires further study, autoimmunity targeting pancreatic *β* cells and their consequential dysfunction is considered the principal cause [4]. The intricate interaction between pathogenic factors and pancreatic cells triggers local inflammation and causes an absolute lack of endogenous insulin secretion *via* islet-specific autoantibodies such as antibodies against insulin (IAA), glutamic acid decarboxylase, and islet cells [5]. The production or detectability of the aforementioned antibodies in the circulation is termed seroconversion. To classify metabolic alterations in the early stages of T1D and to predict its progression, a staging taxonomy for assessing the status of seroconversion, glycemia, and symptomatic manifestation is recommended [1]. After the onset of islet autoimmunity (Stage 1), the disease processes under the uninterrupted loss of *β* cells function through a presymptomatic stage of injured glucose intolerance (Stage 2) and a symptomatic stage, which is featured by clinical symptoms such as polyuria, polydipsia, weight loss, diabetic ketoacidosis, and others (Stage 3).

Heterogeneous risk factors of T1D include genetic susceptibility of polymorphisms within the human leukocyte antigen (HLA) and non-HLA regions, and environmental factors contribute to the progression of T1D, which are still inadequately formulated [2, 6]. Understanding how genetic factors interact with perplexing environmental factors such as nutrition, athletics, infection (especially gut microbiome), and gestational situation, and their relationship with islet autoantibodies remains a challenge and focus of recent research [7, 8].

For a better interpretation of the pathogenesis, progression, and prognosis of T1D, metabolic changes have emerged as an ideal target because of their sensitive response to altered homeostasis and their potential to be clinical biomarkers [9–11]. Moreover, the progression of T1D from seroconversion of islet autoantibodies to ultimate symptomatic stages (or even complicated stages) remains inconclusive from months to decades, with variable rates [1, 2, 5]. Previous cohort studies of genetically susceptible children reported that children who progressed to T1D or developed islet autoimmunity had distinct metabolite profiles in plasma, serum, and cord blood samples compared to the controls [8, 12–15]. Based on the effective biomarkers, it would be feasible to predict the progression and prognosis of T1D at earlier stages, even before the appearance of islet autoantibodies, and to depict its metabolic characteristics throughout the disease cause [16]. Therefore, targeted and/or untargeted metabolomics studies using mass spectrometry (MS) and/or nuclear magnetic resonance (NMR) spectroscopy techniques have been considered robust approaches to identify the dynamic metabolic perturbations and to explore potential diagnostic and prognostic biomarkers for T1D patients [17].

Recently, metabolomic and lipidomic studies based on either longitudinal cohort or cross-sectional case-control cohort designs have focused on T1D children. In contrast to the unclarified causal correlation between metabolic markers and disease progression in case–control studies where a single time-point approach is taken, longitudinal cohort studies provided deeper insights into valuable biomarkers for the interpretation of disease mechanisms and long-term prediction [18, 19]. Many reviews summarized the latest advancements in prediabetes and diabetes metabolomics. Yet, there is still a lack of systematic summaries for childhood T1D, particularly in a manner of integrating metabolic profiling time points [5, 10, 11, 20].

In this review, the metabolic profiling time points of T1D in children are categorized into four dimensions related to the aforementioned stages of the disease. In addition, we applied an effect-score rule and metabolomic analysis to significant or well-pronounced metabolite changes reported in the published studies. We aim to provide a comprehensive clarification and prediction for T1D in children based on the metabolomic methodology during the last 15 years.

## 2. Methods

### 2.1. Searching and Filtering Metabolomics and Lipidomics Publications

Metabolomic and lipidomic studies of T1D children published in English during the last 15 years (from January 1, 2006) were retrieved *via* PubMed (https://www.pubmed.ncbi.nlm.nih.gov) and Web of Science (https://www.webofknowledge.com) by searching keywords, including “*metabolo*^*∗*^” AND “diabetes mellitus, type 1,” “*metabono*^*∗*^” AND “diabetes mellitus, type 1,” OR “*lipido*^*∗*^” AND “diabetes mellitus, type 1.” Publications of clinical trials investigating metabolic changes in T1D children were filtered on ClinicalTrials.gov (https://www.clinicaltrials.gov), using keywords “type 1 diabetes” and “metabolic disorders.” Publications were excluded if they were (1) reviews, meta-analyses, comments, editorials, letters, or other types of nonobservational studies; (2) nonhuman, basic, or technological studies; (3) studies about other types of diabetes, such as type 2 diabetes, gestational diabetes, or prediabetes; (4) studies related to complications, therapies or interventions of diabetes; or (5) studies based on adult subjects, or nonblood samples like urine, stool, earwax, or saliva.

### 2.2. Assessment of Metabolomics and Lipidomics Studies

After retrieval, included publications were full-text reviewed. Significant changes of metabolites and lipids, such as phosphatidylcholines (PCs), triacylglycerols, sphingomyelins (SMs), and amino acids, in islet autoantibody-positive subjects (IA), progressors to T1D (PT1D), and T1D compared to matched control groups were recorded, including their sample types, time points of changes and subjects. An effect-score rule was applied to further assess metabolic perturbations over the development of T1D quantitatively: (1) lipids and metabolites with remarkable changes in case versus control groups were categorized according to the sample types, time points, and subjects. (2) An increase or decrease of lipids and metabolites in specific subjects from individual studies was counted as “+1” or “−1,” respectively, and changes discovered in the same subjects were excluded. (3) A final effect score was calculated for each lipid or metabolite, which was clustered and plotted by R software (version 3.6.3, R Foundation for Statistical Computing, Vienna, Austria), according to their chemical structure and physiological functions, with combinations of some adjacent time points. (4) Pathway analysis was carried out on the metabolome platform in our laboratory and several acknowledged metabolome websites, such as the Human Metabolome Database (https://www.hmdb.ca), PubChem (https://www.pubchem.ncbi.nlm.nih.gov), KEGG (Kyoto Encyclopedia of Genes and Genomes) (https://www.kegg.jp), and MetaboAnalyst 5.0 (https://www.metaboanalyst.ca).

## 3. Results

### 3.1. An Overview of Metabolomics and Lipidomics Studies on Pediatric T1D in the Last 15 Years

**Figure 1:**
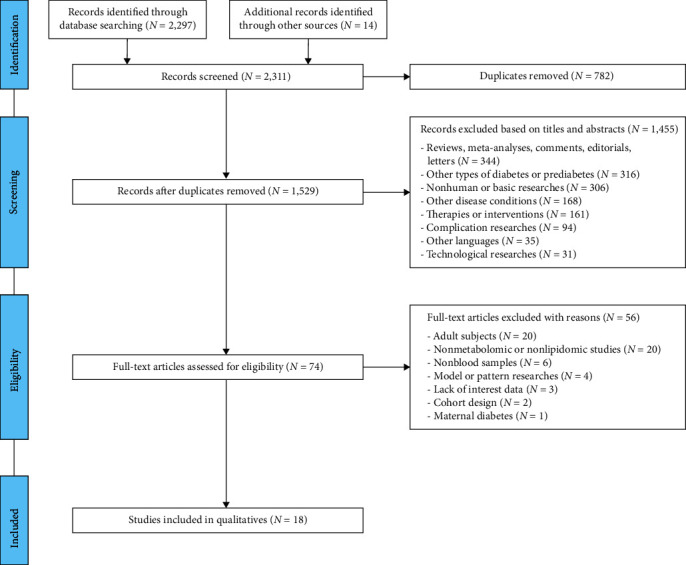
Flowchart of searching and filtering metabolomics and lipidomics studies on childhood T1D.

A total of 2,311 publications were retrieved using the aforementioned searching strategy, of which 782 duplicates were removed, and 1,455 publications were excluded based on titles and abstracts. The remaining 74 publications were full-text assessed, and 56 of them were excluded with reasons as listed (Figure 1). Finally, 18 publications related to metabolic perturbations in childhood T1D were included in the following quantitative assessment (Table 1). These 18 studies were designed to describe changes of metabolites or lipids in the blood samples from either pediatric IA, PT1D groups, or T1D children under different conditions, including positive detection of autoantibodies, diagnosis of T1D, and good or poor glycemic control [8, 12–15, 21–33]. Among them, six were targeted at metabolic disorders under different glycemic control conditions to reflect the management effect, possible complications, and prognosis of T1D [21–23, 26, 31, 33]. Unlike studies from No.14 to No.18, No.1 to No.13 are based on five established cohorts (Table 2). BABYDIAB, DAISY, DIPP, MIDIA, and TEDDY were longitudinal observational cohorts obtaining samples from children at regular intervals to evaluate metabolic perturbations over time [34–39]. DiPiS study was a matched case–control study that aimed to reveal correlations between gestational events, *β*-cell immunity, and childhood T1D [40]. The Danish Remission Phase Study was a prospective observational study that focused on disclosing whether islet autoantibodies or metabolic profiles can predict loss of *β*-cell function during follow-up [41].

### 3.2. Integrated Metabolic Changes during the Development of T1D

**Figure 2:**
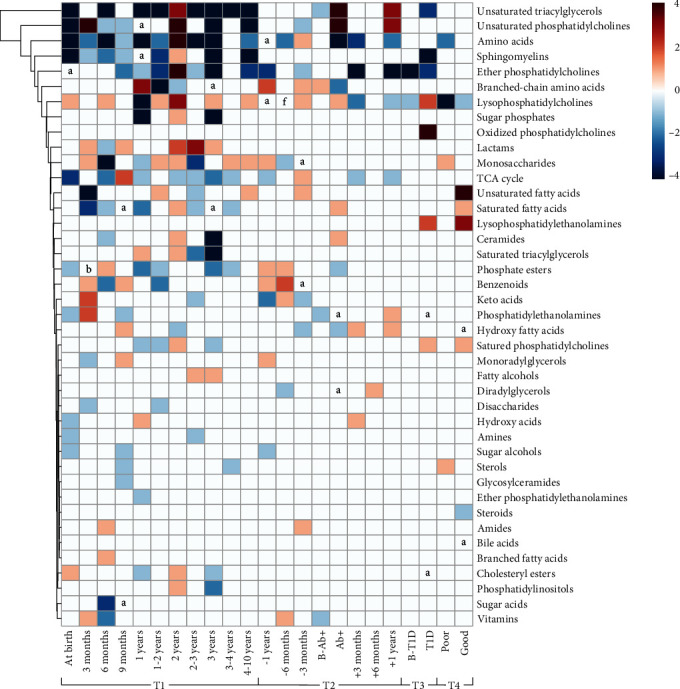
Heatmap of metabolite changes during the development of childhood T1D. Note: Four different time dimensions were included: T1—subjects' ages, from birth to 10 years old; T2—autoimmunity stages, within 1 year before seroconversion, at seroconversion, and within 1 year after seroconversion; T3—before or at diagnosis of T1D; T4—poor or good glycemic control status. Time points from “at birth” to “10 years” refer to the age of patients from birth to 10 years old. Time points from “−1 year” to “+1 year” refer to within 1 year before and after seroconversion. B-Ab^+^: before seroconversion. A-Ab^+^: after seroconversion. B-T1D: before the diagnosis of T1D. T1D: diagnosed as T1D. Poor: poor glycemic control. Good: good glycemic control. TCA acids: metabolites involved in the tricarboxylic acid cycle (or citric acid cycle, Krebs cycle). The label “a” to “f”: refers to inconsistent changes of metabolites due to the same number of increasing and decreasing changes summarized in the publications, with a number pair from 1 to 6.

A total of 293 compounds containing metabolites and lipids that demonstrated significant changes between three case groups, including IA, PT1D, and T1D, and healthy controls were documented, and the samples, time points, and subjects were also summarized (Table S1). Based on the chemical structures and physiological functions, lipids were classified into 16 clusters, including SMs, saturated or unsaturated PCs, and triacylglycerols, and metabolites, were classified into 25 clusters (Table S2). The heatmap plotted a full metabolic profile of IA, PT1D, and T1D groups (Figure 2 and Table S3), with some adjacent time points combined to simplify and consolidate observed characteristics. Individual metabolite change was also shown for a detailed description of the pathogenesis of T1D (Table S4).

Four-time dimensions are characterized throughout follow-up to interpret the progressive spectrum of this disease. The first time dimension 1 (T1) is based on the subjects' ages which range from birth to 10 years old and demonstrates diverse age-related metabolic patterns between the case and control groups. Notably, the included publications revealed consistent signs of high-risk T1D subjects at birth (Figure 2). For instance, unsaturated triacylglycerols (TGs) and PCs, metabolites involved in the tricarboxylic acid (TCA) cycle, SMs, and amino acids distinctively decreased. Phosphate esters, phosphatidylethanolamines (PEs), hydroxy acids, amines, and sugar alcohols might decrease. During growth, metabolic clusters demonstrated specific changing patterns. Several clusters including unsaturated TGs and PCs, metabolites involved in the TCA cycle, amino acids, SMs, and ether PCs revealed a significant decreasing pattern. The cluster of lysophosphatidylcholines (LPCs) and lactams revealed an increasing pattern. In addition, changes of unsaturated TGs, amino acids, SMs, and ether PCs at 2 years of age, unsaturated PCs at 3 months and 2 years of age, lactams at 6 months of age, and LPCs at 1 year of age were distinct from adjacent time points. These might be due to selection bias of significant rather than all potential metabolite changes among included references.

The second time dimension (T2) is related to the seroconversion of subjects ranging from 1 year before seroconversion, at seroconversion, to 1 year after seroconversion, to illuminate metabolic fluctuations involved in islet autoimmunity. Remarkably, several metabolite clusters increased at seroconversion, including unsaturated TGs and PCs, saturated fatty acids, LPCs, and ceramides. And changes in LPCs, branched-chain amino acids (BCAAs), and phosphate esters illustrated a specific pattern associated with the appearance of islet autoantibodies. Contents of these clusters were elevated before seroconversion and lowered after autoantibodies appearance, opposite to changes of hydroxy fatty acids and unsaturated TGs and PCs. It supports the hypothesis that islet immunity responds to intrinsic metabolic disturbance and that once autoantibodies begin to respond, individuals' metabolic homeostasis will partly return to normal [13]. In addition, an increasing pattern of benzenoids appeared before the seroconversion of islet autoantibodies. Ether PCs revealed an obvious decreasing pattern, and metabolites involved in the TCA cycle and amino acids demonstrated a general decreasing trend.

The third time dimension (T3) related to diagnosis was intended to decipher the metabolic profile during the progression to overt T1D. Ether PCs maintained the same decreasing pattern after the appearance of autoantibodies, whereas LPCs showed the reverse pattern. Several clusters, such as oxidized PCs and lysophosphatidylethanolamines (LPEs), saturated PCs, and LPCs revealed increasing levels at the diagnosis of T1D. Unsaturated TGs, SMs, and ether PCs demonstrated the opposite changes. Notably, cluster integration of changes of cholesteryl esters and PEs among the selected studies showed no significant result which still needs to be clarified.

And the fourth time dimension (T4) is based on glycemic control status for assessing whether poor or good glycemic control has effects on metabolic homeostasis. Notably, the clusters of monosaccharides and sterols increased under poor glycemic control. And the clusters of amino acids and LPCs remained decreasing patterns. Under good glycemic control, the unsaturated fatty acids and LPEs demonstrated increasing levels.

### 3.3. Metabolic Pathway Analysis during the Development and Progression of T1D

**Figure 3:**
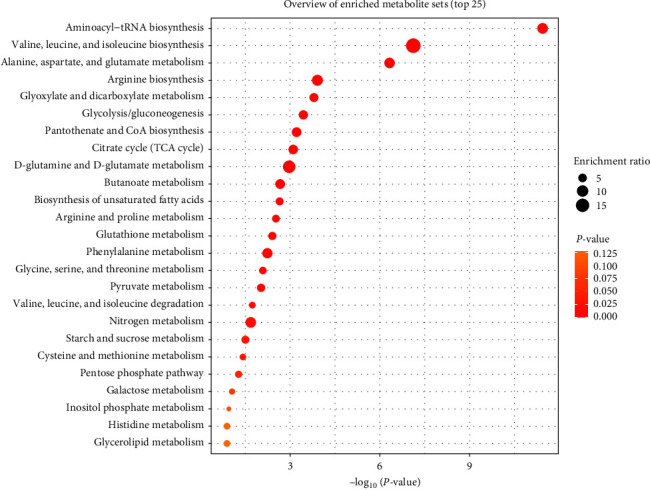
Pathway analysis of differential metabolites during the pathogenesis and progression of childhood T1D.

We also performed the metabolic pathway enrichment analysis to investigate the differential metabolic pathways involved in the pathogenesis and progression of childhood T1D (Figure 3 and Table S5). During the progression of T1D, the significantly changed metabolites and lipids were involved in the biosynthesis of energy metabolism and utilization, such as amino acids, saccharides, organic acids, and fatty acids. Among them, the significantly enriched pathways include the biosynthesis of aminoacyl-tRNA, BCAAs (including valine, leucine, and isoleucine), alanine, aspartate, and glutamate metabolism, arginine biosynthesis, glyoxylate, and dicarboxylate metabolism, glycolysis/gluconeogenesis, pantothenate and CoA biosynthesis, TCA cycle, and D-glutamine and D-glutamate metabolism.

## 4. Discussion

Using biological samples to conduct metabolomic analysis for the composition and contents of metabolites enables a full mapping of the metabolic profile, which responds to the fluctuation of homeostasis [19]. The sensitivity of this methodology reflects homeostatic perturbations in the short term. Continuous and dynamic monitoring will demonstrate the features of metabolic diseases. But it remains controversial whether the metabolome can predict forward disease development independently. A previous study found fewer disease risk markers in the cord blood samples (taken at birth) of late-onset T1D children (children developing islet autoantibodies older than 4 years of age) compared with early-onset T1D children [26]. However, more studies were inclined to the view that in genetically susceptible individuals, early detectable metabolic changes can predict or highly relate to the later progression to T1D [27–29]. In this study, we defined four-time dimensions, the first three relating to disease development and the last relating to its management, to identify specific biomarkers and clarify how short-term metabolic changes correlate with the disease occurrence and further development.

### 4.1. Consensus Biomarkers of the Development of Islet Autoimmunity and Childhood T1D

Several clusters were considered consensus biomarkers of childhood T1D based on synthesizing the metabolomic and lipidomic changes in the 18 studies included (Figure 2). Prominently, choline-containing lipid clusters, including PCs and SMs, possess a predictive potential for prediabetes state for demonstrating a decreasing pattern from birth in the case groups. In prediabetic nonobese diabetic mice, researchers identified a reduction of PC species compared to healthy C57BL/6 mice [42]. This change may imply the deficiency of choline in T1D progressors [27]. As a precursor for the biosynthesis of PCs and SMs, choline is a constituent of phospholipid cellular membranes and an epigenetic regulator of methylation [43, 44]. Furthermore, previous research in asthma patients proved exogenous supplementary choline as an anti-inflammatory factor inducing lowered levels of pro-inflammatory cytokines, including IL-4, IL-5, and TNF-*α*, which are also involved in T1D [45, 46]. Under anaerobic conditions, gut microorganisms encoding the genes of a phospholipase D enzyme will hydrolyze PC to release choline and generate trimethylamine (TMA), known as a disease-associated microbial activity [47]. Degradation of choline into TMA by gut microbiota caused higher levels of trimethylamine *N*-oxide (TMAO) but lower plasma PCs and SMs [48]. TMAO is confirmed as a strong risk marker in diabetes and its related complications [49]. Researchers demonstrated that TMAO induces inflammatory effects through NF-*κ*B and G-protein-coupled receptors (GPCR) signaling [50]. Fewer PCs restrain the absorption of TGs [51], partially interpreting the decreasing pattern of TGs in the plotted heatmap (Figure 2). In this review, LPCs present opposite changes compared to PCs. LPC originates from the cleavage of PC by phospholipase A2 [52]. It remains controversial how LPC affects the development of diabetes. Some studies considered that LPCs' elevation correlates with increased oxidative stress [13], depression of insulin secretion, and activation of homocysteine-induced insulin resistance [52]. However, others found that LPC stimulates the secretion of insulin through binding GPCR and inducing Ca (2+) signaling pathway [53, 54]. Therefore, choline-related lipids demonstrated consistent changes that are expected to be predictive biomarkers of childhood T1D.

Moreover, the change in BCAAs containing valine, leucine, and isoleucine was also remarkable. As essential amino acids and microbial metabolites in the human body, they are intake from the diet and produced by gut microbiota. The cluster of BCAAs was observed to elevate before the seroconversion but decrease after the seroconversion (Figure 2). Consistent with the previous studies, this specific shift prompts the involvement of BCAAs in the pathogenesis of T1D and ascertains their role as a strong predictor for developing diabetes later [10, 11, 55]. In the pathway analysis, the biosynthesis of BCAAs is enriched significantly in the case group (Figure 3). Present studies focus more on the relationship between BCAAs and insulin resistance. Intervention studies in humans and rodents have shown the association between the elevation of BCAAs and insulin resistance [56]. But whether the alterations of BCAAs in insulin sensitivity are a cause or a consequence of homeostatic regulation remains uncertain [57]. It was reported that the activation of the mammalian target of rapamycin by BCAAs suppresses insulin receptor substrate signaling [58]. Other studies believed that insulin resistance results in elevated levels of BCAAs [57]. However, the mechanisms of BCAAs affecting T1D and how they interact with islet autoantibodies are unclarified.

Besides the above metabolites, amino acids and metabolites in the TCA cycle (i.e., citric acid, succinic acid, and alpha-ketoglutaric acid) could also reveal later progression to T1D. They showed a general decreasing trend throughout the seroconversion and occurrence of T1D (Figure 2). Perturbed amino acids and metabolites in the TCA cycle in T1D imply impaired nutrient metabolism, which has been proven to contribute to diabetic cardiovascular autonomic neuropathy, one of the chronic diabetic autonomic neuropathies [59]. Notably, the pathways involved in nutrient metabolism revealed significant changes before the disease onset or even at birth, confirming the view that early metabolic perturbations correlate with later progression to T1D [27–29].

Proline and piperidone demonstrated distinctive changing trends throughout age-related time points (Figure 2). Paradoxically, this study's decreasing trend of proline is inconsistent with the adult and rodent studies of diabetic neuropathy in T1D [59, 60].

**Figure 4:**
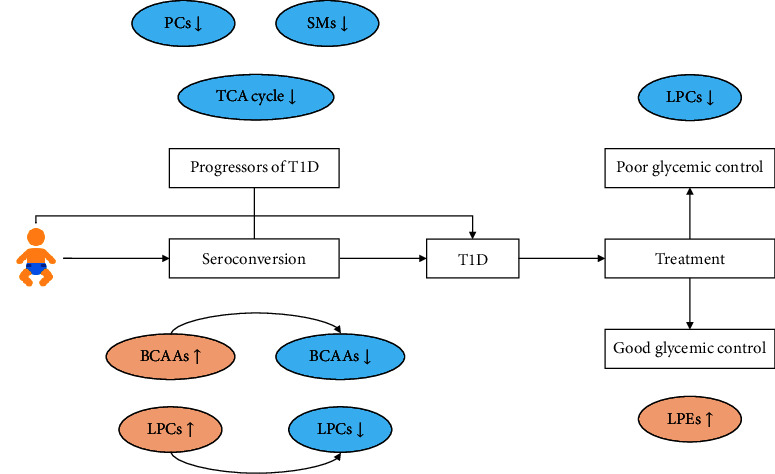
Consensus biomarkers of the development of islet autoimmunity and childhood T1D and the effect of glycemic control on the prognosis.

To conclude, we identified several consensus biomarkers, such as choline-related lipids (including PCs, SMs, and LPCs), BCAAs, amino acids, and metabolites involved in the TCA cycle, potentially predicting the development of islet autoimmunity and T1D (Figure 4). Current research partly explored the mechanism of these metabolites involved in the diabetes progression, but more studies are needed to shed light on the pathogenesis of autoimmunity-induced T1D.

### 4.2. Effects of Glycemic Control on the Prognosis of T1D in Children

Furthermore, we investigate characteristic metabolites of T1D prognosis under different glycemic controls. Increased monosaccharides and sterols were noticed under poor glycemic control. In comparison, LPCs and amino acids demonstrated decreasing trends after seroconversion or progression to overt T1D (Figures 2 and 4). Saturated and unsaturated fatty acids, LPEs, and saturated PCs were revealed at higher levels in T1D patients with good glycemic control (Figure 4). However, the steroids were slightly lower under favorable conditions. Remarkably, LPCs maintained a decreasing trend though it was milder than poor glycemic control. Unsaturated fatty acids are involved in the pathogenesis of T1D, confirmed to correlate with diabetic complications [61].

It is believed that long-term diabetic complications still occur under good glycemic control, and inconsistency of metabolic profiling between the case and control groups may account for it [22]. Therefore, further studies are needed to explore the metabolic features under different glycemic control and their relations with disease prognosis.

### 4.3. Affecting Factors of Islet Autoimmunity and Metabolic Fingerprints

T1D is a heterogeneous autoimmune disease resulting from diverse pathogens, genetic susceptibility, and multiple perplexing environmental factors [2]. Several factors should be considered to better depict metabolic fingerprints of the pathogenesis of autoimmunity and its progression to the disease onset. Individual characteristics, including genetic susceptibility, age of subjects, appearance time of seroconversion, varieties, and numbers of islet autoantibodies, age at the diagnosis of childhood T1D, duration of the disease, and glycemic control may result in the stratified metabotypes. Most islet autoantibodies are confirmed to appear before 1 year of age, then decline from 2 to 5 years of age, and rise again during puberty, with specific characteristics at different ages [28]. The types of positive islet autoantibodies are related to the development of T1D. Subjects detected with two or more autoantibodies are more likely to develop T1D compared to subjects with one autoantibody [2]. Thus, it is understandable that subjects are grouped according to their characteristics, which enables researchers to elaborate more detailed metabolic fingerprints in T1D [15, 24, 26, 30, 62].

Furthermore, extrinsic factors, including gestational environment, infection, diet and nutrition, and medications, are capable of affecting metabolic fingerprints. Metabolic profiling in the samples from cord blood and venous blood obtained at birth is impacted by late maternal pregnancy when the correlation between the offspring metabolism and gestational factors like gestational age and infection is demonstrated [26, 29]. Infection, induced by internal exposure and gut microbiome interaction, is associated with an increased risk of T1D [63]. Previous metabolomic research provides insights into the correlation between disease pathogenesis and diet and nutrition, discovering that the duration of breast milk intake and the diet variety during infancy impact metabolite changes [14, 24]. Diet and nutrition may also mask T1D-associated metabolic signatures during individual development [28]. Therefore, the dieting state of children should be considered when collecting samples to clarify the diet-metabolic pattern [28]. Moreover, some medications may improve T1D progression; for instance, probiotics intake before 28 days of age lowers the risk of T1D for subjects with IAA appearing as the first positive autoantibody [64].

### 4.4. Considerations when Establishing a T1D Cohort Study

Several large cohort studies have been established in countries with a relatively high incidence of T1D (Table 2). An obvious advantage of pediatric T1D cohort study over adult T1D is that establishment of birth cohorts enables much earlier clarification of the correlation between islet autoantibodies and T1D progression. Most of these birth cohorts are longitudinal studies with some common designs, recruiting a large pediatric population with genetic susceptibility from birth or before birth and following up at certain intervals, mostly every 3 months, to obtain samples and to assess islet autoimmunity and disease onset [34–39]. Multiple samples in birth cohort studies containing large datasets would lay a robust foundation for integrated analysis of metabolic signatures, thus better elucidating how genetic risks and environmental factors jointly affect individual metabolism and disease development [19]. Also, cohort studies aiming at clarifying how maternal and intrauterine factors affect the development of offspring T1D have been established, such as DiPiS, a maternal-offspring case–control matched cohort study [40].

When establishing a pediatric T1D cohort study, a refined design is necessary to ensure the accuracy and reliability of its database. Concentrated subject enrollment among several regions or units clarifies how homogeneous genetic susceptibility and specific lifestyle or other region-related factors act on the distribution feature of T1D. It is recommended to establish a relatively larger subject database with matched controls and to obtain multiple samples, especially those noninvasive, for a comprehensive understanding of metabolic perturbations pathologically. In addition, metadata, including body mass index (BMI), feeds and diet, medication history, and clinical test results, provide opportunities for exploring the relationship between environmental factors and integral development. Importantly, elaborate follow-ups, timely tests for islet autoantibodies, and continuous monitoring for diabetic complications are crucial basics for a well-rounded cohort study. Finally, elucidating metabolic changes within different groups remains the core of the entire study.

Moreover, it would be more feasible to launch an observational study for the already diagnosed patients, such as the Danish Remission Phase Study [41]. This type of study unquestionably offers insights into the management and prognosis of T1D more conveniently and viably. However, it is limited to clarifying the causality between metabolic changes and pathogenic conditions.

### 4.5. Predictive Models of Islet Autoimmunity and T1D in Childhood

Recently, regression modeling has been frequently used to establish a prediction model for islet autoimmunity or disease progression in childhood T1D, as summarized in Table 3 [27, 29, 65]. Advanced analysis methodology like integrative machine learning is also applied for establishing a disease prediction model [7, 66]. There are differences among the feature candidates; some are based on metabolomics data, while some integrate data from clinical information and multiomics, including genome, transcriptomics, proteomics, and metabolomics. Given the interaction of heterogeneous risk factors with the unclarified pathogenesis of T1D, it would be wiser to integrate multisource data of subjects [20].

### 4.6. Future Directions

Despite the continuous advancements in understanding the pathogenesis of childhood T1D, efficient serum markers that can reflect or predict *β*-cell function, stress, and disease progress are still lacking [16]. Yet metabolomic and lipidomic discoveries provide comprehensive values, such as clarifying pathogenesis and progression of autoimmunity and T1D; predicting and preventing disease onset, progression, and occurrence of complications at early stages; and directing and assessing the management in terms of diet and potential drugs. However, birth cohorts with precise study designs and better analytical approaches for vast datasets with the potential to greatly enhance our perception of islet autoimmunity and T1D are still needed. Furthermore, metabolomics and lipidomics offer apparent browsing of pathological courses during the development of disease conditions instead of exhaustive underlying mechanisms about how a specific metabolite plays a role in childhood T1D. Therefore, further studies in human, animal, or cell cultures are needed to investigate the mechanisms of metabolites inducing the pathogenesis and complications of T1D.

## 5. Conclusion

In summary, metabolomics and lipidomics studies have proven to be robust tools for elaborating metabolic profiling under the interaction of endogenous predisposition and exogenous effects in the development of childhood T1D. A total of 18 publications in this field during the last 15 years were reviewed, and the metabolic profile of 41 lipid and metabolite clusters of case subjects were mapped based on a tentative effect-score rule. This integrated analysis of metabolomic datasets provides a full-course perspective for understanding the pathogenesis and progression of T1D. Choline-related lipids (including PCs, SMs, and LPCs), BCAAs, and metabolites in the TCA cycle are identified as predictive consensus biomarkers of T1D. Established cohort studies and prediction statistic models of pediatric T1D were also summarized to give a comprehensive view of metabolomic advancements. Still, detailed and reliable metabolomic research aiming at the development of T1D is needed.

## Figures and Tables

**Table 1:** Included metabolomic and lipidomic publications for quantitative assessment.

No.	Year	First author	Journal	Cohort study	Subjects	Number of subjects (*n*)	Samples
1	2011	Pflueger	Diabetes	BABYDIAB	Children of mothers or fathers with T1D	Antibody positive (*n* = 35); antibody negative (*n* = 35)	Serum samples

2	2013	La Torre	Diabetes	DiPiS	Index children diagnosed with T1D before 8 years of age; healthy control subjects matched for HLA risk, sex, date of birth, and the mother's age and gestational age	T1D (*n* = 76); CTR (*n* = 76)	Cord blood samples

3	2008	Oresic	Journal of Experimental Medicine	DIPP	Children progressed to T1D; controls who remained nondiabetic and permanently autoantibody negative	PT1D (*n* = 56); CTR (*n* = 73)	Serum samples, cord blood samples

4	2013	Oresic	The Review of Diabetic Studies	DIPP	Infants later developed T1D; infants developed different numbers of islet autoantibodies during the follow-up; controls matched for sex, HLA-DQB1 genotype, city of birth, and period of birth	PT1D (*n* = 33); 3-4 Aabs (*n* = 31); 2 Aabs (*n* = 31); 1 Aab (*n* = 48); CTR (*n* = 143)	Cord blood samples

5	2018	Lamichhane	Scientific Reports	DIPP	Children progressed to T1D; children developed at least a single islet autoantibody but did not progress to T1D during the follow-up; matched controls	PT1D (*n* = 40); P1Ab (*n* = 40); CTR (*n* = 40)	Plasma samples

6	2019	Lamichhane	Diabetologia	DIPP	Progressors to T1D; children tested positive for at least one antibody in a minimum of two consecutive samples but did not progress to clinical T1D during the follow-up; control children remained islet autoantibody-negative during the follow-up	PT1D (*n* = 40); P1Ab (*n* = 40); CTR (*n* = 40)	Plasma samples

7	2019	Lamichhane	Biomolecules	DIPP	Children progressed to T1D; children who developed at least one islet autoantibody but did not progress to T1D during the follow-up; age-matched controls	PT1D (*n* = 30); P1Ab (*n* = 33); CTR (*n* = 38)	Cord blood samples

8	2020	Sen	Diabetologia	DIPP	Children progressed to T1D; children seroconverted to ≥1 islet autoantibody without progressing to T1D; children remained autoantibody negative during follow-up	PT1D (*n* = 34); P1Ab (*n* = 27); CTR (*N* = 10)	Peripheral blood mononuclear cells (PBMCs)

9	2016	Jørgenrud	Pediatric Diabetes	MIDIA	Age 3 months from case children developed islet autoimmunity and autoantibody negative control children with the HLA DR4-DQ8/DR3-DQ2 genotype	PT1D (*n* = 29); CTR (*n* = 29)	Plasma samples

10	2019	Johnson	Scientific Reports	TEDDY	Cases of confirmed autoantibody positivity to insulin (IAA) or GAD (GADA); matched controls	IA (*n* = 352); CTR	Plasma samples

11	2020	Li	Diabetes	TEDDY	Cases of confirmed autoantibody positivity to insulin (IAA) or GAD (GADA); matched controls	IA (*n* = 414); CTR (*n* = 1234)	Blood samples

12	2021	Li	Diabetes	TEDDY	Cases of confirmed autoantibody positivity to insulin (IAA) or GAD (GADA); matched controls	IA (*n* = 418); CTR (*n* = 1254)	Plasma samples

13	2010	Sorensen	Clinical Biochemistry	–	Patients recently diagnosed with T1D; healthy control individuals	T1D (*n* = 10); CTR (*n* = 10)	Plasma samples, serum samples

14	2013	la Marca	Nutrition and Diabetes	–	Children who developed T1D during the first 6 years of life; controls	PT1D (*N* = 50); CTR (*N* = 200)	Dried blood spot samples

15	2013	Balderas	Electrophoresis	–	Children diagnosed with T1D under good glycemic control (ranging from 6 to 11 years old); healthy controls	T1D (*n* = 34); CTR (*n* = 15)	Plasma samples, urine samples

16	2013	Akmurzina	Journal of Analytical Chemistry	–	Children with different stages of T1D (<1 year, >1 year); controls	T1D (<1 year, *n* = 16); T1D (>1 year, *n* = 20); CTR (*n* = 12)	Plasma samples

17	2017	Bervoets	Diabetology and Metabolic Syndrome	–	Children with poorly controlled T1D; nondiabetic controls aged 8–18 years	T1D (*n* = 7); CTR (*n* = 7)	Plasma samples

18	2019	Semova	Journal of Clinical Lipidology	–	Adolescent subjects with T1D (mean age 15.2 years, mean duration of diabetes 8.2 years) and without diabetes (mean age 15.4 years)	T1D (*n* = 175); CTR (*n* = 74)	Serum samples

T1D, type 1 diabetes; CTR, controls; PT1D, children progressed to T1D during the follow-up; Aab(s), children developed different islet autoantibodies during the follow-up; P1Ab, children developed at least a single islet autoantibody but did not progress to T1D during the follow-up; IA, children with confirmed autoantibody positivity.

**Table 2:** Established cohort studies of childhood T1D in the included metabolomic and lipidomic publications.

No.	Cohort study	Center location	Launch time	Recruitment	Study type	Subject enrollment	Number	Groups	Age	Genetic risk/family history	Samples	Visit design
1	BABYDIAB	Germany	1989	12 years	Longitudinal cohort study	With parents diagnosed as T1D or with mothers diagnosed with gestational diabetes	1,650 Children	Children progressing to T1D during follow-up; children developing autoantibodies at different onset times; controls	From birth to 20 years of age	Having parents with T1D or mothers with gestational diabetes	Cord blood, blood	Venous blood samples were obtained from children at study visits scheduled at 9 months and 2, 5, 8, 11, 14, 17, and 20 years of age

2	Diabetes Autoimmunity Study in the Young (DAISY)	Denver, Colorado, USA	1993	7 years	Longitudinal cohort study	First-degree relatives of patients with T1D and general population children with HLA DR-DQ genotypes	2,547 Children	Children developed T1D; children developed persistent IA and were islet autoantibody-positive at their last study visit; control	From birth to a mean of 4 years (9 months to 9 years of age)	First-degree relatives of patients with T1D (FDRs) and general population children with T1D-susceptibility HLA DR-DQ genotypes	Cord blood, blood	Blood samples were obtained every 3–6 months from birth. Diet data were collected at 3, 6, 9, 12, and 15 months of age

3	Type 1 Diabetes Prediction and Prevention Study (DIPP)	Turku, Oulu in Finland	1994	6 years	Longitudinal cohort study	Born in three centers; at increased genetic risk	Enrollment: 31,526; follow-up: 4,651	Children progressing to T1D during follow-up; children developing only one autoantibody; children developing two autoantibodies; children developing three or four autoantibodies; controls	From birth to 15 years of age	With HLA-DQB1 genotypes ^*∗*^02/ ^*∗*^0302 and ^*∗*^0302/*x* (x is not ^*∗*^02,^*∗*^0301, ^*∗*^0602)	Cord blood, blood, stool	In Turku: monitored at 3-month intervals until 2 years of age and then twice a year; In Oulu and tampere: at the ages of 3, 6, 12, 18, and 24 months and then once a year

4	Environmental Causes of Type 1 Diabetes (MIDIA)	Norway	2001	7 years	Longitudinal cohort study	With a high genetic risk for T1D	Genotyping: around 47, 000; follow-up: more than 900	Children progressing to T1D during follow-up; children developing autoantibodies with different numbers; controls	From birth to 15 years of age	With a high genetic risk for T1D	Blood	Blood samples and questionnaires were obtained at the age of 3, 6, 9, and 12 months, and then annually
5	The Environmental Determinants of Diabetes in the Young Study (TEDDY)	USA, Finland, Sweden, Germany	2007	5 years	Longitudinal cohort study	Younger than 4 months; with specific HLA genotypes, or FDRs of T1D patients	Screening: 3,61,588; enrollment: 17,804; follow-up: over 7,800	Children progressing to T1D during follow-up; children having a different initial autoantibody (IAA-first, GADA-first, IA–2A) with different onset time; children developing more than one autoantibody during follow-up (mAb+); controls	From birth to 15 years of age	With HLADR-DQ haplogenotypes for the risk of T1D; from families with first-degree relatives diagnosed as T1D	Blood, stool, tap water, toenail clippings	From 2 to 14 weeks and 25–28 weeks of the pregnancy and delivery; every 3 months for the first 4 years and until 15 years

6	Diabetes Prediction in Skåne (DiPiS)	Skåne region of Southern Sweden	2000	5 years	Case–control matched cohort study	At increased risk for type 1 diabetes and islet autoantibody appearance	Screening: 33,682 mothers, more than 35,000 newborns	Children progressing to T1D at different times during follow-up; controls	From birth to 10 years of age	At increased risk for type 1 diabetes and islet autoantibody appearance	Cord blood	Every 4–12 months, depending on the number of islet autoantibodies

7	The Danish Remission Phase Study	Denmark	2004	3 years	Prospective observational study	Children and adolescents aged less than 17 years newly diagnosed with T1D	129 children and adolescents	Children and adolescents diagnosed with T1D	12 months after diagnosis	Not mentioned	Blood	Blood samples were collected prospectively 1, 3, 6, and 12 months after diagnosis

**Table 3:** Predictive models of progression to islet autoimmunity or T1D based on metabolomics and lipidomics.

Model	Author	Year	Cohort	Samples	Time horizons	Feature candidates	Aims	Methodologies
1	Webb-Robertson	2021	TEDDY	Plasma samples	(1) 0 months (the time at which positive autoantibodies were first detected for cases); (2) 3 months before IA; (3) 6 months before IA	42 predictors (containing 16 SNPs, 4 traditional risk factors, 11 metabolites, and 11 lipids)	Predict the development of autoimmunity in increased genetic risk TEDDY participants	Metabolomics and lipidomics; integrative machine learning

2	Frohnert	2020	DAISY	Serum samples	T1, earliest available sample before the development of islet autoantibodies (typically age 9–15 months); T2, just before the development of the first autoantibody; T3, just after the development of the first autoantibody; T4, just before a diagnosis of diabetes or the most recent sample for AbPos subjects (islet autoantibody-positive at their last study visit) at the time of sample selection	IA: 16 predictors (containing 3 metadata, 3 SNPs, 6 proteins, and 4 metabolites) T1D: 16 predictors (containing 1 metadata, 3 SNPs, 7 proteins, and 5 metabolites)	Predict IA and progression to T1D	Multiomics; integrative machine learning

3	Lamichhane	2019	DIPP	Cord blood samples	At birth	5 lipids	Predict the risk of progression to T1D	Lipidomics; logistic regression modeling

4	Overgaard	2018	The Danish Remission Phase Study	Plasma samples	1, 3, 6, and 12 months after diagnosis of T1D	3 lipids (at 1 month after diagnosis); Total CE (at 6 months after diagnosis)	Predict residual *β*-cell function	Lipidomics; linear regression modeling

5	Oresic	2013	DIPP	Cord blood samples	At birth	IA: 7 lipids T1D: 7 lipids	Predict IA and progression to T1D at birth	Lipidomics; logistic regression modeling

## Abbreviations

T1D: Type 1 diabetes
IAA: Antibody against insulin
GAD: Glutamic acid decarboxylase
ICA: Islet cell antibody
DKA: Diabetic ketoacidosis
HLA: Human leukocyte antigen
MS: Mass spectrometry
NMR: Nuclear magnetic resonance
IA: Islet autoantibodies-positive subjects
PT1D: Progressors to T1D
KEGG: Kyoto Encyclopedia of Genes and Genomes
DAISY: Diabetes Autoimmunity Study in the Young
DIPP: Type 1 diabetes prediction and prevention study
MIDIA: Environmental causes of type 1 diabetes
TEDDY: The Environmental Determinants of Diabetes in the Young study
DiPiS: Diabetes prediction in skåne
TGs: Triacylglycerols
PCs: Phosphatidylcholines
TCA: Tricarboxylic acid
SMs: Sphingomyelins
PEs: Phosphatidylethanolamines
Ether PCs: Ether phosphatidylcholines
LPCs: Lysophosphatidylcholines
BCAAs: Branched-chain amino acids
Oxidized PCs: Oxidized phosphatidylcholines
LPEs: Lysophosphatidylethanolamines
NOD: Nonobese diabetic
TMA: Trimethylamine
TMAO: Trimethylamine N-oxide
GPCR: G-protein-coupled receptors
BMI: Body mass index.

## Supplementary Materials

Supplementary Materials Table S1: Summary of metabolomic and lipidomic changes in IA, PT1D, or T1D children throughout the follow-up in the included publication archive for the last 15 years. Table S2: Classified clusters of documented lipids and metabolites based on their chemical structures and physiological functions. Table S3: Labeling clusters according to the tentative effective-score rule. Table S4: Metabolite profiling throughout the occurrence and progress of T1D based on the quantitative effect-score rule. Table S5: Pathway analysis of involved metabolites and lipids based on the MetaboAnalyst platform.
