# Supplementary material for: Metabolomics and Lipidomics Studies in Pediatric Type 1 Diabetes: Biomarker Discovery for the Early Diagnosis and Prognosis
Source: Pediatr Diabetes. 2023 Jul 13;2023:6003102. doi: 10.1155/2023/6003102 (PMC12016713; doi:10.1155/2023/6003102)
Supplement: Supplementary Materials — Table S1: Summary of metabolomic and lipidomic changes in IA, PT1D, or T1D children throughout the follow-up in the included publication archive for the last 15 years. Table S2: Classified clusters of documented lipids and metabolites based on their chemical structures and physiological functions. Table S3: Labeling clusters according to the tentative effective-score rule. Table S4: Metabolite profiling throughout the occurrence and progress of T1D based on the quantitative effect-score rule. Table S5: Pathway analysis of involved metabolites and lipids based on the MetaboAnalyst platform. [file 6003102.f1.pdf]

**Supplementary Table 1. Summary of metabolomic and lipidomic changes in IA, PT1D, or T1D children throughout the follow-up in the included publication archive for the last 15 years**

| Sample | Super Class | Class | Sub Class | Direct Parent | Structure/Function Family | Metabolite | Compound | Change | Time Point | Subject | Association | Reference | Notes |
|---|---|---|---|---|---|---|---|---|---|---|---|---|---|
| serum sample | lipids | glycerophospholipids | glycerophosphocholines | phosphatidylcholines | | PC | PC(14:0/18:2) | - | during 6-7yr, during 8-9 yr | 56 PT1D children compared to 73 matched controls | | Oresic M, 2008 | |
| serum sample | lipids | glycerophospholipids | glycerophosphocholines | phosphatidylcholines | | PC | PC(18:2/16:1) | - | during 0-1yr, during 6-7yr, during 7-8yr, during 8-9yr | 56 PT1D children compared to 73 matched controls | | Oresic M, 2008 | |
| serum sample | lipids | glycerophospholipids | glycerophosphocholines | phosphatidylcholines | | PC | PC(18:2/18:2) | - | during 6-7 yr | 56 PT1D children compared to 73 matched controls | | Oresic M, 2008 | |
| serum sample | lipids | glycerophospholipids | glycerophosphocholines | phosphatidylcholines | | PC | PC(16:0/20:5) | - | during 6-7 yr, during 7-8 yr | 56 PT1D children compared to 73 matched controls | | Oresic M, 2008 | |
| serum sample | lipids | glycerophospholipids | glycerophosphocholines | phosphatidylcholines | | PC | PC(18:2/20:4) | - | during 7-8 yr, during 9-10 yr | 56 PT1D children compared to 73 matched controls | | Oresic M, 2008 | |
| serum sample | lipids | glycerophospholipids | glycerophosphocholines | phosphatidylcholines | | PC | PC(38:7) | - | during 6-7yr, during 7-8yr, during 8-9yr | 56 PT1D children compared to 73 matched controls | | Oresic M, 2008 | |
| serum sample | lipids | glycerophospholipids | glycerophosphocholines | phosphatidylcholines | | PC | PC(18:0/22:6) | - | during 6-7yr, during 7-8yr, during 8-9yr, during 9-10 yr | 56 PT1D children compared to 73 matched controls | | Oresic M, 2008 | |
| serum sample | lipids | glycerophospholipids | glycerophosphocholines | phosphatidylcholines | | PC | PC(30:0), PC(32:0), PC(32:1), PC(32:2), PC(34:2), PC(36:4), PC(36:5) | + | at diagnosis (within 14 days) | 10 patients recently diagnosed as T1D compared to 10 matched controls | | Sorensen CM, 2010 | |
| serum sample | lipids | glycerophospholipids | glycerophosphocholines | phosphatidylcholines | | PC | PC(34:0), PC(38:3), PC(38:4), PC(38:5), PC(40:4), PC(40:5) | - | at diagnosis (within 14 days) | 10 patients recently diagnosed as T1D compared to 10 matched controls | | Sorensen CM, 2010 | |
| serum sample | lipids | glycerophospholipids | glycerophosphocholines | phosphatidylcholines | | PC | PC(34:3), PC(36:4), PC(36:5), PC(38:5), PC(40:7) | + | at seroconversion | 35 autoantibody-positive children compared to 35 matched controls | | Pflueger M, 2011 | |
| serum sample | lipids | glycerophospholipids | glycerophosphocholines | phosphatidylcholines | | PC | PC(36:4), PC(38:5), PC(40:7) | + | 1 year after seroconversion | 35 autoantibody-positive children compared to 35 matched controls | | Pflueger M, 2011 | |
| plasma sample | lipids | glycerophospholipids | glycerophosphocholines | phosphatidylcholines | | PC | PC(17:0/2:0) | + | under good glycemic control | 34 T1D children under good glycemic control compared to 15 controls | | Balderas C, 2013 | |
| plasma sample | lipids | glycerophospholipids | glycerophosphocholines | phosphatidylcholines | | PC | PC(32:2), PC(34:3), PC(36:2), PC(36:4), PC(36:5), PC(37:4), PC(38:3), PC(40:4) | + | at 3 months of age | 18 P1Ab children compared to 18 matched controls | | Lamichhane S, 2018 | |
| plasma sample | lipids | glycerophospholipids | glycerophosphocholines | phosphatidylcholines | | PC | PC(33:0) | - | at 18 months of age | 25 PT1D children compared to 26 matched controls | | Lamichhane S, 2018 | |
| cord blood sample | lipids | glycerophospholipids | glycerophosphocholines | phosphatidylcholines | | PC | PC(18:2/18:2) | - | at birth | 15 PT1D children compared to 24 matched controls | | Oresic M, 2008 | |
| cord blood sample | lipids | glycerophospholipids | glycerophosphocholines | phosphatidylcholines | | PC | PC(16:0/18:1), PC(18:0/18:1), PC(18:0/20:3), PC(16:0/18:3), PC(16:0/16:1) | - | at birth | 33 PT1D children compared to matched controls (predominantly seen in 23 T1D progressors who seroconverted before age 4 years) | | Oresic M, 2013 | |
| cord blood sample | lipids | glycerophospholipids | glycerophosphocholines | phosphatidylcholines | | PC | | - | at birth | T1D diagnosed before 4 years of age in 75 index children compared to matched controls | with diagnosis age | La Torre D, 2013 | not included |
| cord blood sample | lipids | glycerophospholipids | glycerophosphocholines | phosphatidylcholines | *unsaturated PCs* | PC | PC(34:3) | - | at 9 months of age, 3 months before seroconversion, at seroconversion | 153 mAb+ cases at 9 months of age, 224 mAb+ cases just prior to seroconversion, 211 mAb+ cases at seroconversion, compared to controls in a nested-case control study | | Johnson RK, 2019 | |
| cord blood sample | lipids | glycerophospholipids | glycerophosphocholines | phosphatidylcholines | | PC | PC(37:3), PC(40:5) | + | at birth | 33 P1Ab children compared to 38 controls | | Lamichhane S, 2019, Biomolecules | |
| PBMCs | lipids | glycerophospholipids | glycerophosphocholines | phosphatidylcholines | | PC | PC(30:0), PC(32:1), PC(35:3), PC(36:3), PC(36:4), PC(38:3), PC(38:4), PC(38:5), PC(40:5) | + | at 24 months of age | 27 P1Ab children compared to 10 controls | | Sen P, 2020 | |
| PBMCs | lipids | glycerophospholipids | glycerophosphocholines | phosphatidylcholines | | PC | PC(32:1), PC(36:4), PC(38:3), PC(38:4), PC(38:5), PC(40:5) | - | at 36 months of age | 27 P1Ab children compared to 10 controls | | Sen P, 2020 | |
| PBMCs | lipids | glycerophospholipids | glycerophosphocholines | phosphatidylcholines | | PC | PC(30:0), PC(38:5) | - | at 12 months of age | 34 PT1D children compared to 10 controls | | Sen P, 2020 | |
| PBMCs | lipids | glycerophospholipids | glycerophosphocholines | phosphatidylcholines | | PC | PC(32:1) | + | at 12 months of age | 34 PT1D children compared to 10 controls | | Sen P, 2020 | |
| PBMCs | lipids | glycerophospholipids | glycerophosphocholines | phosphatidylcholines | | PC | PC(35:3) | - | at 24 months of age | 34 PT1D children compared to 10 controls | | Sen P, 2020 | |
| PBMCs | lipids | glycerophospholipids | glycerophosphocholines | phosphatidylcholines | | PC | PC(35:3) | + | at 36 months of age | 34 PT1D children compared to 10 controls | | Sen P, 2020 | |
| PBMCs | lipids | glycerophospholipids | glycerophosphocholines | phosphatidylcholines | | PC | PC(30:0), PC(32:1), PC(36:3), PC(36:4), PC(38:3), PC(38:4), PC(38:5), PC(40:5) | - | at 36 months of age | 34 PT1D children compared to 10 controls | | Sen P, 2020 | |
| serum sample | lipids | glycerophospholipids | glycerophosphocholines | phosphatidylcholines | | LPC | PC(18:0/0:0) | + | during 0-1yr, during 1-2yr | 56 PT1D children compared to 73 matched controls | | Oresic M, 2008 | |
| serum sample | lipids | glycerophospholipids | glycerophosphocholines | phosphatidylcholines | | LPC | PC(18:3/0:0) | + | during 8-9yr | 56 PT1D children compared to 73 matched controls | | Oresic M, 2008 | |
| serum sample | lipids | glycerophospholipids | glycerophosphocholines | phosphatidylcholines | | LPC | LPC(14:0), LPC(18:2), LPC(18:3), LPC(20:5), LPC(22:6) | + | at diagnosis (within 14 days) | 10 patients recently diagnosed as T1D compared to 10 matched controls | | Sorensen CM, 2010 | |
| serum sample | lipids | glycerophospholipids | glycerophosphocholines | phosphatidylcholines | | LPC | LPC(18:0), LPC(20:3), LPC(22:0) | - | at diagnosis (within 14 days) | 10 patients recently diagnosed as T1D compared to 10 matched controls | | Sorensen CM, 2010 | |
| serum sample | lipids | glycerophospholipids | glycerophosphocholines | phosphatidylcholines | | LPC | PC(18:2/0:0) | - | during 18 and 9 months before seroconversion, during seroconversion and 9 months after seroconversion, during 9 and 18 months after seroconversion, during 27 and 18 months before diagnosis | 56 PT1D children compared to 73 matched controls | | Oresic M, 2008 | |
| serum sample | lipids | glycerophospholipids | glycerophosphocholines | phosphatidylcholines | | LPC | PC(18:2/0:0) | - | during seroconversion of 4 Abs and 9 months after seroconversion, during 9 and 18 months after seroconversion of 4 Abs, during 9 months before seroconversion and seroconversion of GADA+ | 56 PT1D children compared to 73 matched controls | | Oresic M, 2008 | |
| serum sample | lipids | glycerophospholipids | glycerophosphocholines | phosphatidylcholines | | LPC | PC(18:1/0:0) | + | during 9 months before seroconversion and seroconversion of IAA+, during 9 months before seroconversion and seroconversion of ICA+ | 56 PT1D children compared to 73 matched controls | | Oresic M, 2008 | |

| | | | | | | | | | | |
|---|---|---|---|---|---|---|---|---|---|---|
| serum sample | lipids | glycerophospholipids | glycerophosphocholines | phosphatidylcholines | LPC | PC(18:1/0:0) | - | during seroconversion of IAA+ and 9 months after seroconversion, during seroconversion of ICA+ and 9 months after seroconversion, during 9 and 18 months after seroconversion after seroconversion of IA-2A+ | 56 PT1D children compared to 73 matched controls | Oresic M, 2008 |
| serum sample | lipids | glycerophospholipids | glycerophosphocholines | phosphatidylcholines | LPC | PC(18:0/0:0) | + | during 18 and 9 months before seroconversion, during 9 months before seroconversion and seroconversion | 56 PT1D children compared to 73 matched controls | Oresic M, 2008 |
| serum sample | lipids | glycerophospholipids | glycerophosphocholines | phosphatidylcholines | LPC | PC(18:0/0:0) | + | during 18 and 9 months before seroconversion before seroconversion of 4 Abs, during 9 months before seroconversion and seroconversion of IAA+, during 9 months before seroconversion and seroconversion of ICA+ | 56 PT1D children compared to 73 matched controls | Oresic M, 2008 |
| serum sample | lipids | glycerophospholipids | glycerophosphocholines | phosphatidylcholines | LPC | LPC(18:3) | + | at seroconversion, 1 year after seroconversion | 35 autoantibody-positive children compared to 35 matched controls | Pflueger M, 2011 |
| plasma sample | lipids | glycerophospholipids | glycerophosphocholines | phosphatidylcholines | LPC | LPC(14:0) | - | under good glycemic control | 34 T1D children under good glycemic control compared to 15 controls | Balderas C, 2013 |
| plasma sample | lipids | glycerophospholipids | glycerophosphocholines | phosphatidylcholines | LPC | LPC(16:0), LPC(18:0), LPC(18:1), LPC(20:0), LPC(20:1), LPC(20:2) | - | 6 months before seroconversion | 43 GADA-first progressors compared to nonprogressors | Li Q, 2021 |
| plasma sample | lipids | glycerophospholipids | glycerophosphocholines | phosphatidylcholines | LPC | LPC(16:0), LPC(18:0), LPC(18:1), LPC(20:0), LPC(20:1), LPC(20:2) | + | 6 months before seroconversion | 68 IAA-first progressors compared to matched controls | Li Q, 2021 |
| plasma sample | lipids | glycerophospholipids | glycerophosphocholines | phosphatidylcholines | LPC | LPC(16:1) | + | under poor glycemic control | 16 children with T1D less than 1 year compared to 12 controls | Akmurzina VA, 2013 |
| plasma sample | lipids | glycerophospholipids | glycerophosphocholines | phosphatidylcholines | LPC | LPC(18:0), LPC(18:2) | - | under poor glycemic control | 16 children with T1D less than 1 year compared to 12 controls | Akmurzina VA, 2013 |
| plasma sample | lipids | glycerophospholipids | glycerophosphocholines | phosphatidylcholines | LPC | LPC(16:1), LPC(16:0), LPC(18:2), LPC(18:1), LPC(18:0) | - | under poor glycemic control | 20 children with T1D more than 1 year compared to 12 controls | Akmurzina VA, 2013 |
| cord blood sample | lipids | glycerophospholipids | glycerophosphocholines | phosphatidylcholines | LPC | LPC(22:6) | + | at birth | 33 P1Ab children compared to 38 controls | Lamichhane S, 2019, Biomolecules |
| PBMCs | lipids | glycerophospholipids | glycerophosphocholines | phosphatidylcholines | LPC | LPC(18:0), LPC(18:2) | - | at 12 months of age | 27 P1Ab children compared to 10 controls | Sen P, 2020 |
| PBMCs | lipids | glycerophospholipids | glycerophosphocholines | phosphatidylcholines | LPC | LPC(18:0), LPC(18:2) | + | at 24 months of age | 27 P1Ab children compared to 10 controls | Sen P, 2020 |
| PBMCs | lipids | glycerophospholipids | glycerophosphocholines | phosphatidylcholines | LPC | LPC(18:0), LPC(18:2) | - | at 12 months of age | 34 PT1D children compared to 10 controls | Sen P, 2020 |
| PBMCs | lipids | glycerophospholipids | glycerophosphocholines | phosphatidylcholines | LPC | LPC(18:0) | + | at 24 months of age | 34 PT1D children compared to 10 controls | Sen P, 2020 |
| PBMCs | lipids | glycerophospholipids | glycerophosphocholines | phosphatidylcholines | LPC | LPC(18:2) | + | at 36 months of age | 34 PT1D children compared to 10 controls | Sen P, 2020 |
| serum sample | lipids | glycerophospholipids | glycerophosphocholines | phosphatidylcholines | oxidized PC | Oxidized PC(1-16:0/2-O-Hydroxy-7:2)/(16:0/7:1), Oxidized PC(16:0/Hydroxy-18:2), Oxidized PC(1-16:0/2-Hydroxy-18:3), Oxidized PC(16:0/Hydroperoxy-18:3), Oxidized PC(1-18:0/2-C7-oxo), Oxidized PC(18:0/Hydroxy-18:3), Oxidized PC(1-20:0/2-C7-oxo) | + | at diagnosis (within 14 days) | 10 patients recently diagnosed as T1D compared to 10 matched controls | Sorensen CM, 2010 |
| serum sample | lipids | glycerophospholipids | glycerophosphocholines | phosphatidylcholines | ether PC | PC(O-18:1/20:4) | - | during 18 and 9 months before seroconversion, during seroconversion and 9 months after seroconversion, during 9 and 18 months after seroconversion | 56 PT1D children compared to 73 matched controls | Oresic M, 2008 |
| serum sample | lipids | glycerophospholipids | glycerophosphocholines | phosphatidylcholines | ether PC | PC(O-18:1/20:4) | - | during 36 and 27 months before diagnosis | 56 PT1D children compared to 73 matched controls | Oresic M, 2008 |
| serum sample | lipids | glycerophospholipids | glycerophosphocholines | phosphatidylcholines | ether PC | PC(O-16:0/20:4) | - | during 18 and 9 months before seroconversion, during seroconversion and 9 months after seroconversion, during 9 and 18 months after seroconversion | 56 PT1D children compared to 73 matched controls | Oresic M, 2008 |
| serum sample | lipids | glycerophospholipids | glycerophosphocholines | phosphatidylcholines | ether PC | PC(O-16:0/20:4) | - | during 36 and 27 months before diagnosis, during 27 and 18 months before diagnosis | 56 PT1D children compared to 73 matched controls | Oresic M, 2008 |
| serum sample | lipids | glycerophospholipids | glycerophosphocholines | phosphatidylcholines | ether PC | PC(O-38:4) | - | during 18 and 9 months before seroconversion, during seroconversion and 9 months after seroconversion, during 9 and 18 months after seroconversion | 56 PT1D children compared to 73 matched controls | Oresic M, 2008 |
| serum sample | lipids | glycerophospholipids | glycerophosphocholines | phosphatidylcholines | ether PC | PC(O-38:4) | - | during 36 and 27 months before diagnosis | 56 PT1D children compared to 73 matched controls | Oresic M, 2008 |

| | | | | | | | | | | | | |
|---|---|---|---|---|---|---|---|---|---|---|---|---|
| serum sample | lipids | glycerophospholipids | glycerophosphocholines | phosphatidylcholines | | ether PC | PC(O-32:1) | - | during 9 months before seroconversion and seroconversion, during seroconversion and 9 months after seroconversion, during 9 and 18 months after seroconversion | 56 PT1D children compared to 73 matched controls | | Oresic M, 2008 |
| serum sample | lipids | glycerophospholipids | glycerophosphocholines | phosphatidylcholines | | ether PC | PC(O-32:1) | - | during 2-3 yr | 56 PT1D children compared to 73 matched controls | | Oresic M, 2008 |
| serum sample | lipids | glycerophospholipids | glycerophosphocholines | phosphatidylcholines | | ether PC | PC(O-34:1) | - | during 4-5 yr, during 6-7yr | 56 PT1D children compared to 73 matched controls | | Oresic M, 2008 |
| serum sample | lipids | glycerophospholipids | glycerophosphocholines | phosphatidylcholines | | ether PC | PC(O-16:0/20:4), PC(O-38:4) | - | during 1-2 yr | 56 PT1D children compared to 73 matched controls | | Oresic M, 2008 |
| serum sample | lipids | glycerophospholipids | glycerophosphocholines | phosphatidylcholines | | ether PC | PC(O-18:1/20:4) | - | during 1-2 yr, during 7-8 yr | 56 PT1D children compared to 73 matched controls | | Oresic M, 2008 |
| plasma sample | lipids | glycerophospholipids | glycerophosphocholines | phosphatidylcholines | | ether PC | PC(P-32:0), PC(O-32:1) | - | at 9 months of age | 153 mAb+ cases compared to controls in a nested-case control study | | Johnson RK, 2019 |
| plasma sample | lipids | glycerophospholipids | glycerophosphocholines | phosphatidylcholines | | ether PC | PC(16:0e/18:2;16:0p/18:1) | + | at diagnosis (within 14 days) | 10 patients recently diagnosed as T1D compared to 10 matched controls | | Sorensen CM, 2010 |
| plasma sample | lipids | glycerophospholipids | glycerophosphocholines | phosphatidylcholines | | ether PC | PC(16:0p/20:1;16:0e/20:2), PC(16:0p/22:2;16:0e/22:3), PC(16:0p/22:4;16:0e/22:5), PC(18:0p/22:4;18:0e/22:5) | - | at diagnosis (within 14 days) | 10 patients recently diagnosed as T1D compared to 10 matched controls | | Sorensen CM, 2010 |
| PBMCs | lipids | glycerophospholipids | glycerophosphocholines | phosphatidylcholines | | ether PC | PC(O-32:0), PC(O-36:5), PC(O-38:4), PC(O-38:5), PC(O-40:5), PC(P-18:0/22:6) | + | at 24 months of age | 27 P1Ab children compared to 10 controls | | Sen P, 2020 |
| PBMCs | lipids | glycerophospholipids | glycerophosphocholines | phosphatidylcholines | | ether PC | PC(O-36:5), PC(O-38:5), PC(P-18:0/22:6) | - | at 36 months of age | 27 P1Ab children compared to 10 controls | | Sen P, 2020 |
| PBMCs | lipids | glycerophospholipids | glycerophosphocholines | phosphatidylcholines | | ether PC | PC(O-36:5), PC(P-18:0/22:6) | - | at 12 months of age | 34 PT1D children compared to 10 controls | | Sen P, 2020 |
| PBMCs | lipids | glycerophospholipids | glycerophosphocholines | phosphatidylcholines | | ether PC | PC(O-40:5) | + | at 12 months of age | 34 PT1D children compared to 10 controls | | Sen P, 2020 |
| PBMCs | lipids | glycerophospholipids | glycerophosphocholines | phosphatidylcholines | | ether PC | PC(O-36:5), PC(P-18:0/22:6) | + | at 24 months of age | 34 PT1D children compared to 10 controls | | Sen P, 2020 |
| PBMCs | lipids | glycerophospholipids | glycerophosphocholines | phosphatidylcholines | | ether PC | PC(O-38:4) or PC(P-38:3) | - | at 24 months of age | 34 PT1D children compared to 10 controls | | Sen P, 2020 |
| PBMCs | lipids | glycerophospholipids | glycerophosphocholines | phosphatidylcholines | | ether PC | PC(O-32:0), PC(O-36:5), PC(O-38:4), PC(O-38:5), PC(O-40:5), PC(P-18:0/22:6) | - | at 36 months of age | 34 PT1D children compared to 10 controls | | Sen P, 2020 |
| plasma sample | lipids | glycerophospholipids | glycerophosphocholines | phosphatidylcholines | | ether PC | PC(O-22:2/22:3), PC(O-36:5), PC(O-40:4) | + | at 3 months of age | 18 P1Ab children compared to 18 matched controls | | Lamichhane S, 2018 |
| cord blood sample | lipids | glycerophospholipids | glycerophosphocholines | phosphatidylcholines | | ether PC | PC(P-16:0/16:0) | - | at birth | 33 PT1D children compared to matched controls (predominantly seen in 23 T1D progressors who seroconverted before age 4 years) | | Oresic M, 2013 |
| cord blood sample | lipids | glycerophospholipids | glycerophosphocholines | phosphatidylcholines | | ether PC | PC(P-18:0/22:6) | + | at birth | 33 P1Ab children compared to 38 controls | | Lamichhane S, 2019, Biomolecules |
| plasma sample | lipids | glycerophospholipids | glycerophosphocholines | phosphatidylcholines | *PC with a higher number of double bonds (>1)* | | | + | at 3 months of age | 15 PT1D children compared to 18 matched controls | | Lamichhane S, 2018 |
| serum sample | lipids | glycerophospholipids | glycerophosphoethanolamines | phosphatidylethanolamines | | PE | PE(36:2) | + | at diagnosis (within 14 days) | 10 patients recently diagnosed as T1D compared to 10 matched controls | | Sorensen CM, 2010 |
| serum sample | lipids | glycerophospholipids | glycerophosphoethanolamines | phosphatidylethanolamines | | PE | PE(38:5) | - | at diagnosis (within 14 days) | 10 patients recently diagnosed as T1D compared to 10 matched controls | | Sorensen CM, 2010 |
| serum sample | lipids | glycerophospholipids | glycerophosphoethanolamines | phosphatidylethanolamines | | PE | PE(34:2), PE(36:4) | + | at 3 months of age | 18 P1Ab children compared to 18 matched controls | | Lamichhane S, 2018 |
| serum sample | lipids | glycerophospholipids | glycerophosphoethanolamines | phosphatidylethanolamines | | PE | PE(34:2) | - | at 9 months of age, at seroconversion | 153 mAb+ cases at 9 months of age, 211 mAb+ cases at seroconversion, compared to controls in a nested-case control study | | Johnson RK, 2019 |
| serum sample | lipids | glycerophospholipids | glycerophosphoethanolamines | phosphatidylethanolamines | | PE | | - | before seroconversion | in 414 IAA and GADA-first cases compared to controls (in a nested-case control study) | | Li Q, 2020 |
| serum sample | lipids | glycerophospholipids | glycerophosphoethanolamines | phosphatidylethanolamines | | PE | PE(34:4e) | + | at seroconversion, 1 year after seroconversion | 35 autoantibody-positive children compared to 35 matched controls | | Pflueger M, 2011 |
| cord blood sample | lipids | glycerophospholipids | glycerophosphoethanolamines | phosphatidylethanolamines | | PE | PE(38:4e) | - | at birth | T1D diagnosed before 4 years of age in 76 index children compared to matched controls | with diagnosis age | La Torre D, 2013 |
| serum sample | lipids | glycerophospholipids | glycerophosphoethanolamines | phosphatidylethanolamines | | LPE | LPE(16:0), LPE(18:2) | + | at diagnosis (within 14 days) | 10 patients recently diagnosed as T1D compared to 10 matched controls | | Sorensen CM, 2010 |
| plasma sample | lipids | glycerophospholipids | glycerophosphoethanolamines | phosphatidylethanolamines | | LPE | LPE(22:6), LPE(O-18:1), LPE(P-16:0) | + | under good glycemic control | 34 T1D children under good glycemic control compared to 15 controls | | Balderas C, 2013 |
| serum sample | lipids | glycerophospholipids | glycerophosphoethanolamines | phosphatidylethanolamines | plasmalogen | ether PE | PE(O-18:1(1Z)/20:4) | - | at 1 year of age | 56 PT1D children compared to 73 matched controls | | Oresic M, 2008 |
| plasma sample | lipids | glycerophospholipids | glycerophosphates | glycerophosphates | glycerol monophosphate | glycerol-2-phosphate | | + | at 3 months of age | 40 PT1D children compared to 40 P1Ab children and 40 matched controls | | Lamichhane S, 2019, Diabetologia |
| PBMCs | lipids | glycerophospholipids | glycerophosphoinositols | phosphatidylinositols | phosphatidylinositols | PI | PI(18:0/20:4) | + | at 24 months of age | 27 P1Ab children compared to 10 controls | | Sen P, 2020 |
| PBMCs | lipids | glycerophospholipids | glycerophosphoinositols | phosphatidylinositols | phosphatidylinositols | PI | PI(18:0/20:4) | - | at 36 months of age | 27 P1Ab children compared to 10 controls | | Sen P, 2020 |
| PBMCs | lipids | glycerophospholipids | glycerophosphoinositols | phosphatidylinositols | phosphatidylinositols | PI | PI(18:0/20:4) | - | at 36 months of age | 34 PT1D children compared to 10 controls | | Sen P, 2020 |
| serum sample | lipids | sphingolipids | phosphosphingolipids | phosphatidylcholines | | SM | SM(d18:1/16:0) | - | during 7-8 yr | 56 PT1D children compared to 73 matched controls | | Oresic M, 2008 |
| serum sample | lipids | sphingolipids | phosphosphingolipids | phosphatidylcholines | | SM | SM(d18:1/23:1), SM(d18:1/25:1) | - | during 6-7 yr | 56 PT1D children compared to 73 matched controls | | Oresic M, 2008 |
| serum sample | lipids | sphingolipids | phosphosphingolipids | phosphatidylcholines | | SM | SM(d18:1/24:0) | - | during 1-2 yr, during 6-7 yr | 56 PT1D children compared to 73 matched controls | | Oresic M, 2008 |

| | | | | | | | | | | | | |
|---|---|---|---|---|---|---|---|---|---|---|---|---|
| serum sample | lipids | sphingolipids | phosphosphingolipids | phosphatidylcholines | | SM | SM(d16:1/24:0), SM(d18:1/18:0), SM(d18:1/18:1), SM(d18:1/18:2), SM(d18:1/20:0), SM(d18:1/20:1), SM(d18:1/24:1), SM(d18:1/24:2), SM(d20:1/22:3) | - | at diagnosis (within 14 days) | 10 patients recently diagnosed as T1D compared to 10 matched controls | | Sorensen CM, 2010 |
| serum sample | lipids | sphingolipids | phosphosphingolipids | phosphatidylcholines | | SM | SM(d41:2) | - | at 9 months of age | 153 mAb+ cases compared to controls in a nested-case control study | | Johnson RK, 2019 |
| serum sample | lipids | sphingolipids | phosphosphingolipids | phosphatidylcholines | | SM | | - | at 3, 6, 12, 18, 24 and 36 months of age | 40 PT1D children compared to 40 P1Ab children and 40 matched controls | | Lamichhane S, 2018 |
| serum sample | lipids | sphingolipids | phosphosphingolipids | phosphatidylcholines | | SM | SM (d36:2) | - | at 6, 18 months of age | 40 PT1D children compared to 40 matched controls | | Lamichhane S, 2018 |
| cord blood sample | lipids | sphingolipids | phosphosphingolipids | phosphatidylcholines | | SM | SM(d18:1/24:1), SM(d18:1/16:0), SM(d18:1/18:0), SM(d18:1/20:0), SM(d18:2/24:1), SM(18:0/24:2), SM(d18:0/24:1), SM(d18:0/20:0) | - | at birth | 33 PT1D children compared to matched controls (predominantly seen in 23 T1D progressors who seroconverted before age 4 years) | | Oresic M, 2013 |
| cord blood sample | lipids | sphingolipids | phosphosphingolipids | phosphatidylcholines | | SM | | - | at birth | T1D diagnosed before 4 years of age in 76 index children compared to matched controls | with diagnosis age | La Torre D, 2013 |
| PBMCs | lipids | sphingolipids | phosphosphingolipids | phosphatidylcholines | | SM | SM(d18:1/24:0), SM(d41:1) | + | at 24 months of age | 27 P1Ab children compared to 10 controls | | Sen P, 2020 |
| PBMCs | lipids | sphingolipids | phosphosphingolipids | phosphatidylcholines | | SM | SM(d18:1/24:0) | - | at 36 months of age | 27 P1Ab children compared to 10 controls | | Sen P, 2020 |
| PBMCs | lipids | sphingolipids | phosphosphingolipids | phosphatidylcholines | | SM | SM(d18:1/24:0) | + | at 12 months of age | 34 PT1D children compared to 10 controls | | Sen P, 2020 |
| PBMCs | lipids | sphingolipids | phosphosphingolipids | phosphatidylcholines | | SM | SM(d18:1/24:0), SM(d41:1) | - | at 36 months of age | 34 PT1D children compared to 10 controls | | Sen P, 2020 |
| cord blood sample | lipids | sphingolipids | phosphosphingolipids | phosphatidylcholines | *Minor SMs* | | SM(d18:0/16:0), SM(d18:0/18:0) | - | at birth | 31 children who developed 2 autoantibodies compared to matched controls | | Oresic M, 2013 |
| serum sample | lipids | sphingolipids | ceramides | ceramides | | ceramide | Cer(d18:1/22:6) | + | at seroconversion | 35 autoantibody-positive children compared to 35 matched controls | | Pflueger M, 2011 |
| serum sample | lipids | sphingolipids | ceramides | ceramides | | ceramide | Cer(d18:1/22:0) | - | during 0-1yr | 56 PT1D children compared to 73 matched controls | | Oresic M, 2008 |
| PBMCs | lipids | sphingolipids | ceramides | ceramides | | ceramide | Cer(d18:1/22:0), Cer(d18:1/23:0) | + | at 24 months of age | 27 P1Ab children compared to 10 controls | | Sen P, 2020 |
| PBMCs | lipids | sphingolipids | ceramides | ceramides | | ceramide | Cer(d18:1/22:0), Cer(d18:1/23:0), Cer(d18:1/24:0) | - | at 36 months of age | 27 P1Ab children compared to 10 controls | | Sen P, 2020 |
| PBMCs | lipids | sphingolipids | ceramides | ceramides | | ceramide | Cer(d18:1/24:0) | - | at 24 months of age | 34 PT1D children compared to 10 controls | | Sen P, 2020 |
| PBMCs | lipids | sphingolipids | ceramides | ceramides | | ceramide | Cer(d18:1/22:0), Cer(d18:1/23:0), Cer(d18:1/24:0) | - | at 36 months of age | 34 PT1D children compared to 10 controls | | Sen P, 2020 |
| plasma sample | lipids | sphingolipids | glycosphingolipids | glycosyl-N-acylsphingosines | *galactosylceramides, or glycosylceramides* | glycosylceramide | GlcCer(d41:1) | - | at 9 months of age | 153 mAb+ cases compared to controls in a nested-case control study | | Johnson RK, 2019 |
| plasma sample | lipids | glycerolipids | diradylglycerols | | *diradylglycerols* | DG | | - | at seroconversion | 211 mAb+ cases compared to controls in a nested-case control study | | Johnson RK, 2019 |
| plasma sample | lipids | glycerolipids | diradylglycerols | | *diradylglycerols* | DG | DG(34:1) | - | 6 months before seroconversion | 43 GADA-first progressors compared to nonprogressors | | Li Q, 2021 |
| plasma sample | lipids | glycerolipids | diradylglycerols | | *diradylglycerols* | DG | DG(34:1) | + | 6 months before seroconversion | 68 IAA-first progressors compared to matched controls | | Li Q, 2021 |
| serum sample | lipids | glycerolipids | diradylglycerols | | *diradylglycerols* | DG | DG(33:5) | + | at seroconversion | 22 late autoantibody-positive children compared to matched controls | | Pflueger M, 2011 |
| serum sample | lipids | glycerolipids | triradylcglycerols | triacylglycerols | | TG | TG(42:0), TG(16:0/18:1/15:0), TG(16:0/18:0/16:0 | - | during 2-3 yr | 56 PT1D children compared to 73 matched controls | | Oresic M, 2008 |
| serum sample | lipids | glycerolipids | triradylcglycerols | triacylglycerols | | TG | TG(18:1/16:0/12:0), TG(46:2) | - | during 8-9 yr | 56 PT1D children compared to 73 matched controls | | Oresic M, 2008 |
| serum sample | lipids | glycerolipids | triradylcglycerols | triacylglycerols | | TG | TG(18:1/14:0/16:0), TG(16:0/16:0/18:1), TG(18:1/18:1/18:1 | - | during 0-1 yr | 56 PT1D children compared to 73 matched controls | | Oresic M, 2008 |
| serum sample | lipids | glycerolipids | triradylcglycerols | triacylglycerols | | TG | TG(16:0/18:2/14:1 | - | during 0-1 yr, during 1-2 yr, during 8-9yr | 56 PT1D children compared to 73 matched controls | | Oresic M, 2008 |
| serum sample | lipids | glycerolipids | triradylcglycerols | triacylglycerols | | TG | TG(16:1/16:1/18:1) | - | during 0-1 yr, during 1-2 yr, during 3-4yr, during 6-7 yr | 56 PT1D children compared to 73 matched controls | | Oresic M, 2008 |
| serum sample | lipids | glycerolipids | triradylcglycerols | triacylglycerols | | TG | TG(16:0/18:1/17:0 | - | during 2-3 yr, during 3-4 yr | 56 PT1D children compared to 73 matched controls | | Oresic M, 2008 |
| serum sample | lipids | glycerolipids | triradylcglycerols | triacylglycerols | | TG | TG(18:1/18:1/15:0 | - | during 1-2 yr, during 2-3 yr, during 3-4 yr | 56 PT1D children compared to 73 matched controls | | Oresic M, 2008 |
| serum sample | lipids | glycerolipids | triradylcglycerols | triacylglycerols | | TG | TG(16:0/18:1/18:0) | - | during 1-2 yr, during 2-3 yr, during 3-4 yr, during 8-9 yr | 56 PT1D children compared to 73 matched controls | | Oresic M, 2008 |
| serum sample | lipids | glycerolipids | triradylcglycerols | triacylglycerols | | TG | TG(18:2/16:0/18:1 | - | during 1-2 yr, during 6-7 yr | 56 PT1D children compared to 73 matched controls | | Oresic M, 2008 |
| serum sample | lipids | glycerolipids | triradylcglycerols | triacylglycerols | | TG | TG(16:0/18:2/18:2 | - | during 0-1 yr, during 1-2 yr, during 6-7 yr | 56 PT1D children compared to 73 matched controls | | Oresic M, 2008 |
| serum sample | lipids | glycerolipids | triradylcglycerols | triacylglycerols | | TG | TG(16:0/18:3/18:2 | - | during 1-2 yr, during 2-3 yr | 56 PT1D children compared to 73 matched controls | | Oresic M, 2008 |
| serum sample | lipids | glycerolipids | triradylcglycerols | triacylglycerols | | TG | TG(17:0/18:1/18:2) | - | during 0-1 yr, during 1-2 yr, during 2-3 yr, during 3-4yr | 56 PT1D children compared to 73 matched controls | | Oresic M, 2008 |
| serum sample | lipids | glycerolipids | triradylcglycerols | triacylglycerols | | TG | TG(18:0/18:1/18:1) | - | during 0-1 yr, during 1-2 yr, during 2-3 yr, during 3-4yr, during 6-7 yr, during 8-9 yr | 56 PT1D children compared to 73 matched controls | | Oresic M, 2008 |
| serum sample | lipids | glycerolipids | triradylcglycerols | triacylglycerols | | TG | TG(18:2/18:1/18:1 | - | during 1-2 yr | 56 PT1D children compared to 73 matched controls | | Oresic M, 2008 |
| serum sample | lipids | glycerolipids | triradylcglycerols | triacylglycerols | | TG | TG(18:1/18:2/18:2), TG(56:6) | - | during 0-1 yr, during 1-2 yr | 56 PT1D children compared to 73 matched controls | | Oresic M, 2008 |
| serum sample | lipids | glycerolipids | triradylcglycerols | triacylglycerols | | TG | TG(46:1), TG(56:2) | + | at diagnosis (within 14 days) | 10 patients recently diagnosed as T1D compared to 10 matched controls | | Sorensen CM, 2010 |
| serum sample | lipids | glycerolipids | triradylcglycerols | triacylglycerols | | TG | TG(50:4), TG(54:4), TG(54:5), TG(54:6), TG(56:7) | - | at diagnosis (within 14 days) | 10 patients recently diagnosed as T1D compared to 10 matched controls | | Sorensen CM, 2010 |
| serum sample | lipids | glycerolipids | triradylcglycerols | triacylglycerols | | TG | TG(55:2), TG(55:4) | + | at seroconversion, 1 year after seroconversion | 35 autoantibody-positive children compared to 35 matched controls | | Pflueger M, 2011 |

| | | | | | | | | | | | | |
|---|---|---|---|---|---|---|---|---|---|---|---|---|
| serum sample | lipids | glycerolipids | triradylcglycerols | triacylglycerols | | TG | TG(16:0/18:1/16:0), TG(50:2), TG(55:3) | + | at seroconversion | 35 autoantibody-positive children compared to matched controls | | Pflueger M, 2011 |
| serum sample | lipids | glycerolipids | triradylcglycerols | triacylglycerols | | TG | TG(49:3) | + | at seroconversion, 1 year after seroconversion | 22 late autoantibody-positive children compared to matched controls | | Pflueger M, 2011 |
| cord blood sample | lipids | glycerolipids | triradylcglycerols | triacylglycerols | | TG | | - | at birth | T1D diagnosed before 4 years of age in 75 index children compared to matched controls, T1D diagnosed before 2 years of age in 75 index children compared to matched controls | with diagnosis age | La Torre D, 2013 |
| cord blood sample | lipids | glycerolipids | triradylcglycerols | triacylglycerols | | TG | TG(46:1), TG(46:2), TG(47:1), TG(48:1) | - | at birth | 30 PT1D children compared to 38 controls | | Lamichhane S, 2019, Biomolecules |
| cord blood sample | lipids | glycerolipids | triradylcglycerols | triacylglycerols | | TG | TG(51:3), TG(58:1) | + | at birth | 30 PT1D children compared to 38 controls | | Lamichhane S, 2019, Biomolecules |
| PBMCs | lipids | glycerolipids | triradylcglycerols | triacylglycerols | | TG | TG(14:0/18:2/18:2), TG(16:0/18:2/18:3), TG(18:1/18:1/18:1), TG(18:2/18:2/18:2), TG(47:2), TG(48:3), TG(50:1), TG(50:3), TG(50:5), TG(51:3), TG(51:4), TG(52:5), TG(54:2), TG(54:3), TG(56:2) | - | at 12 months of age | 27 P1Ab children compared to 10 controls | | Sen P, 2020 |
| PBMCs | lipids | glycerolipids | triradylcglycerols | triacylglycerols | | TG | TG(18:0/18:0/18:0 | + | at 12 months of age | 27 P1Ab children compared to 10 controls | | Sen P, 2020 |
| PBMCs | lipids | glycerolipids | triradylcglycerols | triacylglycerols | | TG | TG(14:0/18:2/18:2), TG(18:1/18:1/18:1), TG(47:2), TG(50:1), TG(50:3), TG(51:4), TG(54:2), TG(54:3), TG(56:2) | + | at 24 months of age | 27 P1Ab children compared to 10 controls | | Sen P, 2020 |
| PBMCs | lipids | glycerolipids | triradylcglycerols | triacylglycerols | | TG | TG(18:0/18:0/18:0), TG(51:1), TG(52:0), TG(52:5), TG(53:2), TG(54:2), TG(56:2) | - | at 36 months of age | 27 P1Ab children compared to 10 controls | | Sen P, 2020 |
| PBMCs | lipids | glycerolipids | triradylcglycerols | triacylglycerols | | TG | TG(14:0/18:2/18:2), TG(16:0/18:2/18:3), TG(18:1/18:1/18:1), TG(47:2), TG(48:3), TG(50:1), TG(50:3), TG(50:5), TG(51:1), TG(51:3), TG(51:4), TG(52:5), TG(54:2), TG(54:3), TG(56:2) | - | at 12 months of age | 34 PT1D children compared to 10 controls | | Sen P, 2020 |
| PBMCs | lipids | glycerolipids | triradylcglycerols | triacylglycerols | | TG | TG(14:0/18:2/18:2), TG(16:0/18:2/18:3), TG(18:2/18:2/18:2), TG(48:3), TG(50:3), TG(51:1), TG(51:3), TG(52:5) | - | at 24 months of age | 34 PT1D children compared to 10 controls | | Sen P, 2020 |
| PBMCs | lipids | glycerolipids | triradylcglycerols | triacylglycerols | | TG | TG(18:0/18:0/18:0), TG(18:1/18:1/18:1), TG(51:4) | + | at 24 months of age | 34 PT1D children compared to 10 controls | | Sen P, 2020 |
| PBMCs | lipids | glycerolipids | triradylcglycerols | triacylglycerols | | TG | TG(14:0/18:2/18:2), TG(16:0/18:2/18:3), TG(18:0/18:0/18:0), TG(18:1/18:1/18:1), TG(18:1/18:2/18:2), TG(18:2/18:2/18:2), TG(50:5), TG(51:1), TG(52:0), TG(52:5), TG(53:2), TG(54:2) | - | at 36 months of age | 34 PT1D children compared to 10 controls | | Sen P, 2020 |
| PBMCs | lipids | glycerolipids | triradylcglycerols | triacylglycerols | | TG | TG(48:3), TG(56:2) | + | at 36 months of age | 34 PT1D children compared to 10 controls | | Sen P, 2020 |
| plasma sample | lipids | glycerolipids | triradylcglycerols | triacylglycerols | *unsaturated TGs* | TG | | - | at seroconversion | 211 mAb+ cases compared to controls in a nested-case control study | | Johnson RK, 2019 |
| plasma sample | lipids | glycerolipids | triradylcglycerols | triacylglycerols | *unsaturated TGs* | TG | | - | before seroconversion | in 414 IAA and GADA-first cases compared to controls (in a nested-case control study) | | Li Q, 2020 |
| cord blood sample | lipids | glycerolipids | triradylcglycerols | triacylglycerols | *long chain poliunsaturated fatty acid-containing TGs* | TG | TG(56:7), TG(56:8), TG(58:8) | - | at birth | 31 children who developed 3-4 autoantibodies compared to matched controls | | Oresic M, 2013 |
| plasma sample | lipids | glycerolipids | monoradylglycerols | 1-monoacylglycerols | | 1-monopalmitin | | - | at 3 months of age | 11 PT1D children compared to 18 matched controls | | Lamichhane S, 2019, Diabetologia |

| | | | | | | | | | | | | |
|---|---|---|---|---|---|---|---|---|---|---|---|---|
| plasma sample | lipids | glycerolipids | monoradylglycerols | 1-monoradylglycerols | | 1-monoolein | | + | at 9 months of age | in IAA-first cases compared to controls (414 cases in a nested-case control study) | with autoantibody | Li Q, 2020 |
| plasma sample | lipids | glycerolipids | monoradylglycerols | 1-monoradylglycerols | | 1-monoolein | | + | 12 months before seroconversion | in GADA-first cases compared to controls (414 cases in a nested-case control study) | with autoantibody | Li Q, 2020 |
| serum sample | lipids | steroids | steroid esters | cholesteryl esters | | CE | CE(20:5) | + | at diagnosis (within 14 days) | 10 patients recently diagnosed as T1D compared to 10 matched controls | | Sorensen CM, 2010 |
| serum sample | lipids | steroids | steroid esters | cholesteryl esters | | CE | CE(20:3) | - | at diagnosis (within 14 days) | 10 patients recently diagnosed as T1D compared to 10 matched controls | | Sorensen CM, 2010 |
| cord blood sample | lipids | steroids | steroid esters | cholesteryl esters | | CE | CE(18:2) | + | at birth | 30 PT1D children compared to 38 controls | | Lamichhane S, 2019, Biomolecules |
| PBMCs | lipids | steroids | steroid esters | cholesteryl esters | | CE | CE(18:1) | + | at 24 months of age | 27 P1Ab children compared to 10 controls | | Sen P, 2020 |
| PBMCs | lipids | steroids | steroid esters | cholesteryl esters | | CE | CE(18:1) | - | at 36 months of age | 27 P1Ab children compared to 10 controls | | Sen P, 2020 |
| PBMCs | lipids | steroids | steroid esters | cholesteryl esters | | CE | CE(18:1) | - | at 12 months of age | 34 PT1D children compared to 10 controls | | Sen P, 2020 |
| plasma sample | lipids | steroids | steroidal glycosides | steroid glucuronide conjugates | deoxycholic acids | deoxycholic acid 3-glucuronide | | + | under good glycemic control | 34 T1D children under good glycemic control compared to 15 controls | with glycemic control | Balderas C, 2013 |
| plasma sample | lipids | steroids | steroidal glycosides | steroid glucuronide conjugates | tetrahydroaldosterone | tetrahydroaldosterone-3-glucuronide | | - | under good glycemic control | 34 T1D children under good glycemic control compared to 15 controls | with glycemic control | Balderas C, 2013 |
| plasma sample | lipids | steroids | bile acids | C24 bile acids | | 3,7,12-trioxochola-1,4-dien-24-oic acid | | - | under good glycemic control | 34 T1D children under good glycemic control compared to 15 controls | with glycemic control | Balderas C, 2013 |
| serum sample | lipids | steroids | ergostane steroids | ergosterols | cholesterol absorption markers | campesterol | | + | under poor glycemic control | 175 T1D adolescents (mean age 15.2 years, mean duration of diabetes 8.2 years) compared to 74 controls (mean age 15.4 years) | with cholesterol metabolism, positively correlated with HbA1c levels | Semova I, 2019 |
| serum sample | lipids | steroids | stigmastanes | stigmastanes | cholesterol absorption markers | beta-sitosterol | | + | under poor glycemic control | 175 T1D adolescents (mean age 15.2 years, mean duration of diabetes 8.2 years) compared to 74 controls (mean age 15.4 years) | with cholesterol metabolism, positively correlated with HbA1c levels | Semova I, 2019 |
| plasma sample | lipids | steroids | cholestane steroids | cholesterols and derivatives | | cholesterol | | - | at 9 months of age | 68 IAA-first case subjects (early IAA) compared to controls | | Li Q, 2021 |
| serum sample | lipids | steroids | cholestane steroids | cholesterols and derivatives | | cholesterol | | - | during 3-4yr | 56 PT1D children compared to 73 matched controls | | Oresic M, 2008 |
| serum sample | lipids | steroids | cholestane steroids | cholesterols and derivatives | cholesterol synthesis markers | lathosterol | | - | under poor glycemic control | 175 T1D adolescents (mean age 15.2 years, mean duration of diabetes 8.2 years) compared to 74 controls (mean age 15.4 years) | with cholesterol metabolism | Semova I, 2019 |
| plasma sample | lipids | steroids | vitamin D and derivatives | vitamin D and derivatives | | 25-hydroxyvitamin D | | - | within 1 year before seroconversion | 171 T1D progressors compared to nonprogressors | | Li Q, 2021 |
| plasma sample | lipids | steroids | vitamin D and derivatives | vitamin D and derivatives | | 25-hydroxyvitamin D | | - | at 6 months of age | 191 IAA-first and GADA-first case subjects compared to controls | with genotype (HLA-DR3/DR4 haplotype and the corresponding alleles, i.e. at HLA genes DRB1, DRQ1, DQB1) | Li Q, 2021 |
| plasma sample | lipids | fatty acyls | fatty acids | | *saturated fatty acids* | | | + | at seroconversion | 211 mAb+ cases compared to controls in a nested-case control study | | Johnson RK, 2019 |
| plasma sample | lipids | fatty acyls | fatty acids | medium-chain fatty acids | | lauric acid | | - | at 3 months of age | in IAA-first cases compared to controls (414 cases in a nested-case control study) | with autoantibody | Li Q, 2020 |
| plasma sample | lipids | fatty acyls | fatty acids | medium-chain fatty acids | | lauric acid | | - | at 30 months of age | in GADA-first cases compared to controls (414 cases in a nested-case control study) | with autoantibody | Li Q, 2020 |
| plasma sample | lipids | fatty acyls | fatty acids | medium-chain fatty acids | | pelargonic acid | | - | at 9 months of age | in IAA-first cases compared to controls (414 cases in a nested-case control study) | with autoantibody | Li Q, 2020 |
| plasma sample | lipids | fatty acyls | fatty acids | medium-chain fatty acids | hydroxy fatty acids | 2-hydroxy capric acid | | - | under good glycemic control | 34 T1D children under good glycemic control compared to 15 controls | with glycemic control | Balderas C, 2013 |
| plasma sample | lipids | fatty acyls | fatty acids | | hydroxy fatty acids | 11,12-dihydroxy arachidic acid | | + | under good glycemic control | 34 T1D children under good glycemic control compared to 15 controls | with glycemic control | Balderas C, 2013 |
| plasma sample | lipids | fatty acyls | fatty acids | long-chain fatty acids | NEFA, saturated fatty acids | palmitic acid | palmitic acid (C16:0) | + | under good glycemic control | 34 T1D children under good glycemic control compared to 15 controls | with glycemic control | Balderas C, 2013 |
| plasma sample | lipids | fatty acyls | fatty acids | long-chain fatty acids | NEFA, saturated fatty acids | palmitic acid | | - | at 3 months of age | 11 PT1D children compared to 18 matched controls | | Lamichhane S, 2019, Diabetologia |
| PBMCs | lipids | fatty acyls | fatty acids | long-chain fatty acids | NEFA, saturated fatty acids | palmitic acid | | + | at 36 months of age | 34 PT1D children compared to 10 controls | | Sen P, 2020 |
| plasma sample | lipids | fatty acyls | fatty acids | long-chain fatty acids | NEFA, unsaturated fatty acids | palmitoleic acid | palmitoleic acid (C16:1 omega 9) | + | under good glycemic control | 34 T1D children under good glycemic control compared to 15 controls | with glycemic control | Balderas C, 2013 |
| plasma sample | lipids | fatty acyls | fatty acids | long-chain fatty acids | NEFA, unsaturated fatty acids | palmitoleic acid | | + | 3 months before seroconversion | in GADA-first cases compared to controls (414 cases in a nested-case control study) | with autoantibody | Li Q, 2020 |
| serum sample | lipids | fatty acyls | fatty acids | long-chain fatty acids | NEFA, unsaturated fatty acids | palmitoleic acid | | + | during 5-6yr | 56 PT1D children compared to 73 matched controls | | Oresic M, 2008 |
| plasma sample | lipids | fatty acyls | fatty acids | long-chain fatty acids | unsaturated fatty acids | oleic acid | oleic acid (C18:1 omega 9) | + | under good glycemic control | 34 T1D children under good glycemic control compared to 15 controls | with glycemic control | Balderas C, 2013 |
| plasma sample | lipids | fatty acyls | fatty acids | long-chain fatty acids | unsaturated fatty acids | oleic acid | | - | at 3 months of age | 11 PT1D children compared to 18 matched controls | | Lamichhane S, 2019, Diabetologia |
| plasma sample | lipids | fatty acyls | fatty acids | long-chain fatty acids | NEFA, straight-chain saturated fatty acid | stearic acid | | - | at 3 months of age | 11 PT1D children compared to 18 matched controls | | Lamichhane S, 2019, Diabetologia |
| plasma sample | lipids | fatty acyls | fatty acids | long-chain fatty acids | eicosanoids, polyunsaturated fatty acid, essential fatty acid | arachidonic acid | | - | at 3 months of age | 11 PT1D children compared to 18 matched controls | | Lamichhane S, 2019, Diabetologia |
| plasma sample | lipids | fatty acyls | fatty acids | long-chain fatty acids | eicosanoids, polyunsaturated fatty acid, essential fatty acid | arachidonic acid | | + | at 15 months of age | in GADA-first cases compared to controls (414 cases in a nested-case control study) | with autoantibody | Li Q, 2020 |
| plasma sample | lipids | fatty acyls | fatty acids | long-chain fatty acids | | pentadecanoic acid | | - | at 6 months of age | 20 PT1D children compared to 28 matched controls | | Lamichhane S, 2019, Diabetologia |
| plasma sample | lipids | fatty acyls | fatty acids | long-chain fatty acids | monounsaturated fatty acids, microbial catabolites | 11-eicosenoic acid | | - | at 3 months of age | 11 PT1D children compared to 18 matched controls | | Lamichhane S, 2019, Diabetologia |
| PBMCs | lipids | fatty acyls | fatty acids | long-chain fatty acids | saturated fatty acids | myristic acid | | - | at 12 months of age | 27 P1Ab children compared to 10 controls | | Sen P, 2020 |
| PBMCs | lipids | fatty acyls | fatty acids | long-chain fatty acids | saturated fatty acids | myristic acid | | - | at 12 months of age | 34 PT1D children compared to 10 controls | | Sen P, 2020 |
| plasma sample | lipids | fatty acyls | fatty acids | very long-chain fatty acids | NEFA, polyunsaturated fatty acid | adrenic acid | adrenic acid (C22:4 omega 6) | + | under good glycemic control | 34 T1D children under good glycemic control compared to 15 controls | with glycemic control | Balderas C, 2013 |
| plasma sample | lipids | fatty acyls | fatty alcohols | fatty alcohols | | 1-dodecanol | | + | at 36 months of age | 26 PT1D children compared to 25 matched controls | | Lamichhane S, 2019, Diabetologia |

| | | | | | | | | | | | | |
|---|---|---|---|---|---|---|---|---|---|---|---|---|
| plasma sample | lipids | fatty acyls | fatty alcohols | long-chain fatty alcohols | | octadecanol | | + | at 33 months of age | in GADA-first cases compared to controls (414 cases in a nested-case control study) | with autoantibody | Li Q, 2020 |
| plasma sample | lipids | fatty acyls | lineolic acids | lineolic acids | NEFA, doubly unsaturated fatty acids | linoleic acid | linoleic acid (C18:2 omega 6) | + | under good glycemic control | 34 T1D children under good glycemic control compared to 15 controls | with glycemic control | Balderas C, 2013 |
| plasma sample | lipids | fatty acyls | lineolic acids | lineolic acids | NEFA, doubly unsaturated fatty acids | linoleic acid | | - | at 3 months of age | 11 PT1D children compared to 18 matched controls | | Lamichhane S, 2019, Diabetologia |
| plasma sample | lipids | fatty acyls | lineolic acids | lineolic acids | NEFA, doubly unsaturated fatty acids | linoleic acid | | - | at 30 months of age | in GADA-first cases compared to controls (414 cases in a nested-case control study) | with autoantibody | Li Q, 2020 |
| plasma sample | lipids | fatty acyls | lineolic acids | lineolic acids | polyunsaturated fatty acid (PUFA) | linolenic acid | linoleinic acid (C18:3 omega 3) | + | under good glycemic control | 34 T1D children under good glycemic control compared to 15 controls | with glycemic control | Balderas C, 2013 |
| plasma sample | lipids | prenol lipids | quinone and hydroquinone lipids | tocopherols | | alpha-tocopherol | | + | 6 months before seroconversion | in IAA-first cases compared to controls (414 cases in a nested-case control study) | with autoantibody | Li Q, 2020 |
| plasma sample | organic acids | carboxylic acids | carboxylic acids | carboxylic acids | | acetic acid | | - | during 3-4yr | 56 PT1D children compared to 73 matched controls | | Oresic M, 2008 |
| plasma sample | organic acids | carboxylic acids | dicarboxylic acids | dicarboxylic acids | *dicarboxylic acids* | adipic acid, TCA cycle (succinic acid, malic acid) | | + | at 9 months of age | 153 mAb+ cases compared to controls in a nested-case control study | | Johnson RK, 2019 |
| cord blood sample | organic acids | carboxylic acids | dicarboxylic acids | dicarboxylic acids | TCA cycle, microbial catabolites | succinic acid | | - | at birth | 15 PT1D children compared to 24 matched controls | | Oresic M, 2008 |
| serum sample | organic acids | carboxylic acids | dicarboxylic acids | dicarboxylic acids | TCA cycle, microbial catabolites | succinic acid | | - | during 0-1yr, during 3-4yr | 56 PT1D children compared to 73 matched controls | | Oresic M, 2008 |
| serum sample | organic acids | carboxylic acids | dicarboxylic acids | dicarboxylic acids | TCA cycle, microbial catabolites | succinic acid | | + | during 9 months before seroconversion and seroconversion | 56 PT1D children compared to 73 matched controls | | Oresic M, 2008 |
| plasma sample | organic acids | carboxylic acids | dicarboxylic acids | dicarboxylic acids | TCA cycle, microbial catabolites | succinic acid | | - | at 6 months of age | 20 PT1D children compared to 28 matched controls | | Lamichhane S, 2019, Diabetologia |
| PBMCs | organic acids | carboxylic acids | dicarboxylic acids | dicarboxylic acids | | glutaric acid | | + | at 24 months of age | 27 P1Ab children compared to 10 controls | | Sen P, 2020 |
| PBMCs | organic acids | carboxylic acids | dicarboxylic acids | dicarboxylic acids | | glutaric acid | | - | at 36 months of age | 27 P1Ab children compared to 10 controls | | Sen P, 2020 |
| cord blood sample | organic acids | carboxylic acids | tricarboxylic acids | tricarboxylic acids | TCA cycle | citric acid | | - | at birth | 15 PT1D children compared to 24 matched controls | | Oresic M, 2008 |
| serum sample | organic acids | carboxylic acids | tricarboxylic acids | tricarboxylic acids | TCA cycle | citric acid | | - | 3 months after seroconversion | 42 children postive for 1 autoantibody compared to matched controls | | Jørgenrud B, 2016 |
| serum sample | organic acids | carboxylic acids | tricarboxylic acids | tricarboxylic acids | TCA cycle | citric acid | | - | during 2-3yr | 56 PT1D children compared to 73 matched controls | | Oresic M, 2008 |
| serum sample | organic acids | carboxylic acids | tricarboxylic acids | tricarboxylic acids | TCA cycle | citric acid | | - | during 9 and 18 months after seroconversion of ICA+, during 18 and 9 months before seroconversion of IA-2A+ | 13 PT1D children compared to 26 matched controls | with autoantibody | Oresic M, 2008 |
| PBMCs | organic acids | carboxylic acids | tricarboxylic acids | tricarboxylic acids | TCA cycle | citric acid | | - | at 12,24,36 months of age | 27 P1Ab children compared to 10 controls | | Sen P, 2020 |
| PBMCs | organic acids | carboxylic acids | tricarboxylic acids | tricarboxylic acids | TCA cycle | citric acid | | - | at 36 months of age | 34 PT1D children compared to 10 controls | | Sen P, 2020 |
| plasma sample | organic acids | carboxylic acids | amino acids | | *amino acids* | | | - | at seroconversion | 211 mAb+ cases compared to controls in a nested-case control study | | Johnson RK, 2019 |
| plasma sample | organic acids | carboxylic acids | amino acids | | *aromatic amino acids* | | | - | at seroconversion | 211 mAb+ cases compared to controls in a nested-case control study | | Johnson RK, 2019 |
| plasma sample | organic acids | carboxylic acids | amino acids | branched fatty acids | | itaconic acid | | + | at 6 months of age | in IAA-first cases compared to controls (414 cases in a nested-case control study) | with autoantibody | Li Q, 2020 |
| PBMCs | organic acids | carboxylic acids | amino acids | branched-chain fatty acids | *BCAA* | | | + | at 12 months of age | 27 P1Ab children compared to 10 controls | | Sen P, 2020 |
| PBMCs | organic acids | carboxylic acids | amino acids | branched-chain fatty acids | *BCAA* | | | + | at 24 months of age | 27 P1Ab children compared to 10 controls | | Sen P, 2020 |
| PBMCs | organic acids | carboxylic acids | amino acids | branched-chain fatty acids | *BCAA* | | | ± | at 12 months of age | 34 PT1D children compared to 10 controls | | Sen P, 2020 |
| PBMCs | organic acids | carboxylic acids | amino acids | branched-chain fatty acids | *BCAA* | | | ± | at 24 months of age | 34 PT1D children compared to 10 controls | | Sen P, 2020 |
| PBMCs | organic acids | carboxylic acids | amino acids | branched-chain fatty acids | *BCAA* | | | ± | at 36 months of age | 34 PT1D children compared to 10 controls | | Sen P, 2020 |
| plasma sample | organic acids | carboxylic acids | amino acids | valine | BCAA, EAA, pyruvate family amino acid, proteinogenic amino acid | valine | | - | at seroconversion | 42 children postive for 1 autoantibody compared to matched controls | | Jørgenrud B, 2016 |
| plasma sample | organic acids | carboxylic acids | amino acids | valine | BCAA, EAA, pyruvate family amino acid, proteinogenic amino acid | valine | | - | at 15,18,21,24 months of age | in IAA-first cases which experienced seroconversion after 2 years of age compared to controls (414 cases in a nested-case control study) | with autoantibody | Li Q, 2020 |
| PBMCs | organic acids | carboxylic acids | amino acids | valine | BCAA, EAA, pyruvate family amino acid, proteinogenic amino acid | valine | | + | at 12 months of age | 27 P1Ab children compared to 10 controls | | Sen P, 2020 |
| PBMCs | organic acids | carboxylic acids | amino acids | valine | BCAA, EAA, pyruvate family amino acid, proteinogenic amino acid | valine | | + | at 12 months of age | 34 PT1D children compared to 10 controls | | Sen P, 2020 |
| serum sample | organic acids | carboxylic acids | amino acids | leucine | BCAA, EAA, pyruvate family amino acid, proteinogenic amino acid | leucine | | + | during 9 months before seroconversion and seroconversion | 56 PT1D children compared to 73 matched controls | | Oresic M, 2008 |
| serum sample | organic acids | carboxylic acids | amino acids | leucine | BCAA, EAA, pyruvate family amino acid, proteinogenic amino acid | leucine | | + | during 18 and 9 months before seroconversion of GADA+ | 13 PT1D children compared to 26 matched controls | with autoantibody | Oresic M, 2008 |
| plasma sample | organic acids | carboxylic acids | amino acids | leucine | BCAA, EAA, pyruvate family amino acid, proteinogenic amino acid | leucine | | - | at seroconversion | 42 children postive for 1 autoantibody compared to matched controls | | Jørgenrud B, 2016 |
| serum sample | organic acids | carboxylic acids | amino acids | isoleucine | BCAA, EAA, aspartate family amino acid, proteinogenic amino acid | isoleucine | | + | before seroconversion | 56 children who progressed to T1D | | Oresic M, 2008 |
| serum sample | organic acids | carboxylic acids | amino acids | isoleucine | BCAA, EAA, aspartate family amino acid, proteinogenic amino acid | isoleucine | | + | during 18 and 9 months before seroconversion of ICA+ | 13 PT1D children compared to 26 matched controls | with autoantibody | Oresic M, 2008 |
| serum sample | organic acids | carboxylic acids | amino acids | isoleucine | BCAA, EAA, aspartate family amino acid, proteinogenic amino acid | isoleucine | | - | at 15,18,21,24 months of age | in IAA-first cases and GADA-first which experienced seroconversion after 2 years of age compared to controls (414 cases in a nested-case control study) | with autoantibody | Li Q, 2020 |
| cord blood sample | organic acids | carboxylic acids | amino acids | glycine | | glycine | | - | at birth | 15 PT1D children compared to 24 matched controls | | Oresic M, 2008 |
| cord blood sample | organic acids | carboxylic acids | amino acids | serine | | serine | | - | at birth | 15 PT1D children compared to 24 matched controls | | Oresic M, 2008 |
| plasma sample | organic acids | carboxylic acids | amino acids | serine | | serine | | - | under poor glycemic control | 7 T1D children compared to 7 matched controls | | Bervoets L, 2017 |
| PBMCs | organic acids | carboxylic acids | amino acids | serine | | serine | | + | at 24 months of age | 27 P1Ab children compared to 10 controls | | Sen P, 2020 |
| PBMCs | organic acids | carboxylic acids | amino acids | serine | | serine | | - | at 36 months of age | 27 P1Ab children compared to 10 controls | | Sen P, 2020 |
| PBMCs | organic acids | carboxylic acids | amino acids | serine | | serine | | + | at 24 months of age | 34 PT1D children compared to 10 controls | | Sen P, 2020 |
| plasma sample | organic acids | carboxylic acids | amino acids | alanine | NEAA, proteinogenic amino acid | alanine | | - | at 12 months of age | in IAA-first cases compared to controls (414 cases in a nested-case control study) | with autoantibody | Li Q, 2020 |
| plasma sample | organic acids | carboxylic acids | amino acids | alanine | NEAA, proteinogenic amino acid | alanine | | - | 6 months before seroconversion | 43 GADA-first progressors compared to nonprogressors | | Li Q, 2021 |

| | | | | | | | | | | | |
|---|---|---|---|---|---|---|---|---|---|---|---|
| plasma sample | organic acids | carboxylic acids | amino acids | alanine | NEAA, proteinogenic amino acid | alanine | - | 12 months before seroconversion | in GADA-first cases compared to controls (414 cases in a nested-case control study) | with autoantibody | Li Q, 2020 |
| serum sample | organic acids | carboxylic acids | amino acids | alanine | NEAA, proteinogenic amino acid | alanine | - | during 5-6yr | 56 PT1D children compared to 73 matched controls | | Oresic M, 2008 |
| serum sample | organic acids | carboxylic acids | amino acids | alanine | NEAA, proteinogenic amino acid | alanine | + | during 9 months before seroconversion and seroconversion | 56 PT1D children compared to 73 matched controls | | Oresic M, 2008 |
| serum sample | organic acids | carboxylic acids | amino acids | alanine | NEAA, proteinogenic amino acid | alanine | - | during 9 months before seroconversion and seroconversion of ICA+ | 13 PT1D children compared to 26 matched controls | with autoantibody | Oresic M, 2008 |
| dried blood spot sample | organic acids | carboxylic acids | amino acids | alanine | NEAA, proteinogenic amino acid | alanine | - | at birth | 50 PT1D children compared to 200 matched controls | | la Marca G, 2013 |
| PBMCs | organic acids | carboxylic acids | amino acids | alanine | NEAA, proteinogenic amino acid | alanine | + | at 24,36 months of age | 27 P1Ab children compared to 10 controls | | Sen P, 2020 |
| PBMCs | organic acids | carboxylic acids | amino acids | alanine | NEAA, proteinogenic amino acid | alanine | + | at 24,36 months of age | 34 PT1D children compared to 10 controls | | Sen P, 2020 |
| plasma sample | organic acids | carboxylic acids | amino acids | alanine | | N-methylalanine | - | 6 months before seroconversion | in GADA-first cases compared to controls (414 cases in a nested-case control study) | with autoantibody | Li Q, 2020 |
| plasma sample | organic acids | carboxylic acids | amino acids | tyrosine | EAA, precursor for adrenalin, erythrose 4-phosphate/phosphoenolpyruvate family amino acid, proteinogenic amino acid | tyrosine | - | at seroconversion | 29 children who were positive for two or three autoantibodies or had progressed to T1D compared to matched controls | | Jørgenrud B, 2016 |
| plasma sample | organic acids | carboxylic acids | amino acids | tyrosine | EAA, precursor for adrenalin, erythrose 4-phosphate/phosphoenolpyruvate family amino acid, proteinogenic amino acid | tyrosine | - | 3 months after seroconversion | 42 children postive for 1 autoantibody compared to matched controls | | Jørgenrud B, 2016 |
| PBMCs | organic acids | carboxylic acids | amino acids | tyrosine | EAA, precursor for adrenalin, erythrose 4-phosphate/phosphoenolpyruvate family amino acid, proteinogenic amino acid | tyrosine | - | at 12 months of age | 34 PT1D children compared to 10 controls | | Sen P, 2020 |
| serum sample | organic acids | carboxylic acids | amino acids | methionine | EAA | methionine | - | at seroconversion, 1 year after seroconversion | 13 autoantibody-positive children with ABs developed at or before 2 years old compared to matched controls | | Pflueger M, 2011 |
| serum sample | organic acids | carboxylic acids | amino acids | methionine | EAA | methionine | + | at 6 months of age | 20 PT1D children compared to 28 matched controls | | Lamichhane S, 2019, Diabetologia |
| serum sample | organic acids | carboxylic acids | amino acids | methionine | EAA | methionine | + | at 3 months of age | 40 PT1D children compared to 40 P1Ab children and 40 matched controls | | Lamichhane S, 2019, Diabetologia |
| serum sample | organic acids | carboxylic acids | amino acids | methionine | EAA | methionine | - | at 6 months of age | in GADA-first cases compared to controls (414 cases in a nested-case control study) | with autoantibody | Li Q, 2020 |
| plasma sample | organic acids | carboxylic acids | amino acids | phenylalanine | EAA, precursor for catecholamines, erythrose 5-phosphate/phosphoenolpyruvate family amino acid, proteinogenic amino acid | phenylalanine | - | at 3 months of age | 42 children postive for 1 autoantibody compared to matched controls | | Jørgenrud B, 2016 |
| PBMCs | organic acids | carboxylic acids | amino acids | phenylalanine | EAA, precursor for catecholamines, erythrose 6-phosphate/phosphoenolpyruvate family amino acid, proteinogenic amino acid | phenylalanine | + | at 24 months of age | 27 P1Ab children compared to 10 controls | | Sen P, 2020 |
| PBMCs | organic acids | carboxylic acids | amino acids | phenylalanine | EAA, precursor for catecholamines, erythrose 7-phosphate/phosphoenolpyruvate family amino acid, proteinogenic amino acid | phenylalanine | - | at 36 months of age | 27 P1Ab children compared to 10 controls | | Sen P, 2020 |
| PBMCs | organic acids | carboxylic acids | amino acids | phenylalanine | EAA, precursor for catecholamines, erythrose 8-phosphate/phosphoenolpyruvate family amino acid, proteinogenic amino acid | phenylalanine | - | at 12,36 months of age | 34 PT1D children compared to 10 controls | | Sen P, 2020 |
| PBMCs | organic acids | carboxylic acids | amino acids | phenylalanine | EAA, precursor for catecholamines, erythrose 9-phosphate/phosphoenolpyruvate family amino acid, proteinogenic amino acid | phenylalanine | + | at 24 months of age | 34 PT1D children compared to 10 controls | | Sen P, 2020 |
| plasma sample | organic acids | carboxylic acids | amino acids | proline | | proline | - | at 3,6,9,12,18,21,24,27,30,33,36 months of age (before seroconversion) | in GADA-first cases which experienced seroconversion before 2 years of age compared to controls (414 cases in a nested-case control study) | with autoantibody | Li Q, 2020 |
| plasma sample | organic acids | carboxylic acids | amino acids | proline | | proline | - | at 30 months of age | in GADA-first cases compared to controls (414 cases in a nested-case control study) | with autoantibody | Li Q, 2020 |
| PBMCs | organic acids | carboxylic acids | amino acids | proline | | proline | + | at 24 months of age | 27 P1Ab children compared to 10 controls | | Sen P, 2020 |
| PBMCs | organic acids | carboxylic acids | amino acids | proline | | proline | + | at 24 months of age | 34 PT1D children compared to 10 controls | | Sen P, 2020 |
| plasma sample | organic acids | carboxylic acids | amino acids | proline | | trans-4-hydroxyproline/L-hydroxyproline | - | at 27 months of age | in GADA-first cases compared to controls (414 cases in a nested-case control study) | with autoantibody | Li Q, 2020 |
| plasma sample | organic acids | carboxylic acids | amino acids | proline | | trans-4-hydroxyproline/L-hydroxyproline | - | at 6 months of age | 20 PT1D children compared to 28 matched controls | | Lamichhane S, 2019, Diabetologia |
| serum sample | organic acids | carboxylic acids | amino acids | proline | | trans-4-hydroxyproline/L-hydroxyproline | - | at seroconversion | 22 autoantibody-positive children with ABs developed at or after 8 years old compared to matched controls | | Pflueger M, 2011 |
| plasma sample | organic acids | carboxylic acids | amino acids | aspartic acid | | aspartic acid | - | at 6 months of age | 20 PT1D children compared to 28 matched controls | | Lamichhane S, 2019, Diabetologia |

| | | | | | | | | | | | |
|---|---|---|---|---|---|---|---|---|---|---|---|
| serum sample | organic acids | carboxylic acids | amino acids | aspartic acid | | aspartic acid | - | during 9 and 18 months after seroconversion of GADA+ | 13 PT1D children compared to 26 matched controls | with autoantibody | Oresic M, 2008 |
| PBMCs | organic acids | carboxylic acids | amino acids | aspartic acid | | aspartic acid | + | at 24 months of age | 27 P1Ab children compared to 10 controls | | Sen P, 2020 |
| PBMCs | organic acids | carboxylic acids | amino acids | aspartic acid | | aspartic acid | + | at 24 months of age | 34 PT1D children compared to 10 controls | | Sen P, 2020 |
| serum sample | organic acids | carboxylic acids | amino acids | alpha amino acids and derivatives | | creatinine | - | at birth | 13 PT1D children compared to 26 matched controls | | Oresic M, 2008 |
| serum sample | organic acids | carboxylic acids | amino acids | L-alpha-amino acids | proteinogenic amino acids | glutamic acid/glutamate | + | during 18 and 9 months before seroconversion of GADA+, during 9 months before seroconversion and seroconversion of IAA+ | 13 PT1D children compared to 26 matched controls | with autoantibody | Oresic M, 2008 |
| serum sample | organic acids | carboxylic acids | amino acids | L-alpha-amino acids | proteinogenic amino acids | glutamic acid/glutamate | + | during 18 and 9 months before seroconversion, during 9 months before seroconversion and seroconversion | 56 PT1D children compared to 73 matched controls | | Oresic M, 2008 |
| plasma sample | organic acids | carboxylic acids | amino acids | L-alpha-amino acids | proteinogenic amino acids | glutamic acid/glutamate | - | at 3,6 months of age | 11 PT1D children compared to 18 matched controls at 3 months of age, 20 PT1D children compared to 28 matched controls at 6 months of age | | Lamichhane S, 2019, Diabetologia |
| plasma sample | organic acids | carboxylic acids | amino acids | L-alpha-amino acids | proteinogenic amino acids | glutamic acid/glutamate | - | at seroconversion | 13 autoantibody-positive children with ABs developed at or before 2 years old compared to matched controls | | Pflueger M, 2011 |
| PBMCs | organic acids | carboxylic acids | amino acids | L-alpha-amino acids | proteinogenic amino acids | glutamic acid/glutamate | + | at 24 months of age | 27 P1Ab children compared to 10 controls | | Sen P, 2020 |
| PBMCs | organic acids | carboxylic acids | amino acids | L-alpha-amino acids | proteinogenic amino acids | glutamic acid/glutamate | + | at 24 months of age | 34 PT1D children compared to 10 controls | | Sen P, 2020 |
| serum sample | organic acids | carboxylic acids | amino acids | L-alpha-amino acids | NEAA | glutamine | - | during seroconversion of 4 Abs and 9 months after seroconversion, during 18 and 9 months before seroconversion of IAA+ | 13 PT1D children compared to 26 matched controls | | Oresic M, 2008 |
| PBMCs | organic acids | carboxylic acids | amino acids | L-alpha-amino acids | EAA, aspartate family amino acid, proteinogenic amino acid | threonine | - | at 12,36 months of age | 27 P1Ab children compared to 10 controls | | Sen P, 2020 |
| plasma sample | organic acids | carboxylic acids | amino acids | L-alpha-amino acids | urea cycle, non-proteinogenic L-alpha-amino acid | ornithine | - | at seroconversion | 29 children who were positive for two or three autoantibodies or had progressed to T1D compared to matched controls | | Jørgenrud B, 2016 |
| plasma sample | organic acids | carboxylic acids | amino acids | L-alpha-amino acids | urea cycle, non-proteinogenic L-alpha-amino acid | ornithine | - | 3 months after seroconversion | 42 children postive for 1 autoantibody compared to matched controls | | Jørgenrud B, 2016 |
| cord blood sample | organic acids | carboxylic acids | amino acids | L-alpha-amino acids | urea cycle, non-proteinogenic L-alpha-amino acid | ornithine | - | at birth | 15 PT1D children compared to 24 matched controls | | Oresic M, 2008 |
| PBMCs | organic acids | carboxylic acids | amino acids | L-alpha-amino acids | urea cycle, non-proteinogenic L-alpha-amino acid | ornithine | + | at 24 months of age | 34 PT1D children compared to 10 controls | | Sen P, 2020 |
| PBMCs | organic acids | carboxylic acids | amino acids | L-alpha-amino acids | | lysine | - | at 12 months of age | 27 P1Ab children compared to 10 controls | | Sen P, 2020 |
| PBMCs | organic acids | carboxylic acids | amino acids | L-alpha-amino acids | | lysine | - | at 12 months of age | 34 PT1D children compared to 10 controls | | Sen P, 2020 |
| plasma sample | organic acids | carboxylic acids | amino acids | alpha-amino acids | | L-5-oxoproline | - | at 3,6 months of age | 11 PT1D children compared to 18 matched controls at 3 months of age, 20 PT1D children compared to 28 matched controls at 6 months of age | | Lamichhane S, 2019, Diabetologia |
| PBMCs | organic acids | carboxylic acids | amino acids | alpha-amino acids | | L-5-oxoproline | - | at 36 months of age | 27 P1Ab children compared to 10 controls | | Sen P, 2020 |
| PBMCs | organic acids | carboxylic acids | amino acids | alpha-amino acids | | L-5-oxoproline | - | at 24,36 months of age | 34 PT1D children compared to 10 controls | | Sen P, 2020 |
| plasma sample | organic acids | carboxylic acids | amino acids | gamma-amino acids | monocarboxylic acid | GABA | + | at 3 months of age | in IAA-first cases compared to controls (414 cases in a nested-case control study) | with autoantibody | Li Q, 2020 |
| serum sample | organic acids | carboxylic acids | amino acids | gamma-amino acids | monocarboxylic acid | GABA | + | during 18 and 9 months before seroconversion of ICA+, GADA+ | 13 PT1D children compared to 26 matched controls | with autoantibody | Oresic M, 2008 |
| plasma sample | organic acids | carboxylic acids | amino acids | cysteine and derivatives | | L-cysteine | - | under poor glycemic control | 7 T1D children compared to 7 matched controls | | Bervoets L, 2017 |
| plasma sample | organic acids | carboxylic acids | amino acids | cysteine and derivatives | | cystine | - | at 12 months of age | in IAA-first cases compared to controls (414 cases in a nested-case control study) | with autoantibody | Li Q, 2020 |
| PBMCs | organic acids | carboxylic acids | amino acids | cysteine and derivatives | | cystine | - | at 12,36 months of age | 27 P1Ab children compared to 10 controls | | Sen P, 2020 |
| PBMCs | organic acids | carboxylic acids | amino acids | cysteine and derivatives | | cystine | - | at 36 months of age | 34 PT1D children compared to 10 controls | | Sen P, 2020 |
| serum sample | organic acids | carboxylic acids | amino acids | histidine and derivatives | | 1-methylhistidine | - | at seroconversion | 13 autoantibody-positive children with ABs developed at or before 2 years old compared to matched controls | | Pflueger M, 2011 |
| plasma sample | organic acids | hydroxy acids | alpha hydroxy acids | alpha hydroxy acids | 3-hydroxypropanoic acid | lactic acid | + | at 12 months of age | in IAA-first cases compared to controls (414 cases in a nested-case control study) | with autoantibody | Li Q, 2020 |
| serum sample | organic acids | hydroxy acids | alpha hydroxy acids | alpha hydroxy acids | 4-hydroxypropanoic acid | lactic acid | + | during seroconversion of ICA+ and 9 months after seroconversion, during seroconversion of IA-2A+ and 9 months after seroconversion | 13 PT1D children compared to 26 matched controls | with autoantibody | Oresic M, 2008 |
| cord blood sample | organic acids | hydroxy acids | alpha hydroxy acids | alpha hydroxy acids | 5-hydroxypropanoic acid | lactic acid | - | at birth | 15 PT1D children compared to 24 matched controls | | Oresic M, 2008 |
| serum sample | organic acids | hydroxy acids | alpha hydroxy acids | alpha hydroxy acids | | 2-hydroxybutyric acid | + | during seroconversion and 9 months after seroconversion of IAA+, during 9 and 18 months after seroconversion of GADA+, during seroconversion and 9 months after seroconversion of ICA+, during seroconversion and 9 months after seroconversion of of IA-2A+ | 13 PT1D children compared to 26 matched controls | with autoantibody | Oresic M, 2008 |
| | organic acids | hydroxy acids | alpha hydroxy acids | alpha hydroxy acids | | 2-hydroxybutyric acid | - | during 9 months before seroconversion and seroconversion of IAA+ | 13 PT1D children compared to 26 matched controls | with autoantibody | Oresic M, 2008 |
| plasma sample | organic acids | hydroxy acids | beta hydroxy acids | beta hydroxy acids | | 3-hydroxybutyric acid | - | at 24 months of age | in IAA-first cases compared to controls (414 cases in a nested-case control study) | with autoantibody | Li Q, 2020 |
| plasma sample | organic acids | hydroxy acids | beta hydroxy acids | beta hydroxy acids | omega-hydroxy fatty acid | 3,4-dihydroxybutanoic acid | - | by seroconversion | in 26 P1Ab children | A-P1Ab to B-P1Ab | Lamichhane S, 2019, Diabetologia |
| plasma sample | organic acids | hydroxy acids | short-chain hydroxy acids | short-chain hydroxy acids | | 2-hydroxyglutaric acid | + | at 9 months of age | in GADA-first cases compared to controls (414 cases in a nested-case control study) | with autoantibody | Li Q, 2020 |
| plasma sample | organic acids | hydroxy acids | short-chain hydroxy acids | short-chain hydroxy acids | omega-hydroxy fatty acid, hydroxybutyric acid | 2,4-dihydroxybutanoic acid | - | at seroconversion | 42 children postive for 1 autoantibody compared to matched controls | | Jørgenrud B, 2016 |
| plasma sample | organic acids | organic phosphoric acids and derivatives | phosphate esters | monoalkyl phosphates | | methylphosphate | - | at 3 months of age | in GADA-first cases compared to controls (414 cases in a nested-case control study) | with autoantibody | Li Q, 2020 |
| plasma sample | organic acids | organic phosphoric acids and derivatives | phosphate esters | phosphoethanolamines | | phosphoethanolamine | + | 12 months before seroconversion | in GADA-first cases compared to controls (414 cases in a nested-case control study) | with autoantibody | Li Q, 2020 |

| | | | | | | | | | | | |
|---|---|---|---|---|---|---|---|---|---|---|---|
| plasma sample | organic acids | organic phosphoric acids | phosphate esters | dicarboxylic acids | | diglycerol | - | at 3 months of age | in IAA-first cases compared to controls (414 cases in a nested-case control study) | with autoantibody | Li Q, 2020 |
| plasma sample | organic acids | organic phosphoric acids | phosphate esters | dicarboxylic acids | | diglycerol | + | at 6 months of age | in GADA-first cases compared to controls (414 cases in a nested-case control study) | with autoantibody | Li Q, 2020 |
| PBMCs | organic acids | organic phosphoric acids and derivatives | phosphate esters | phosphate esters | | phosphoenolpyruvic acid | - | at 12,36 months of age | 27 P1Ab children compared to 10 controls | | Sen P, 2020 |
| PBMCs | organic acids | organic phosphoric acids and derivatives | phosphate esters | phosphate esters | | phosphoenolpyruvic acid | - | at 12,36 months of age | 34 PT1D children compared to 10 controls | | Sen P, 2020 |
| serum sample | organic acids | keto acids | short-chain keto acids | short-chain keto acids | | ketoleucine/2-ketoisocaproic acid | - | during 18 and 9 months before seroconversion of IAA+, during 9 months before seroconversion and seroconversion of IAA+ | 56 PT1D children compared to 73 matched controls | with autoantibody | Oresic M, 2008 |
| plasma sample | organic acids | keto acids | short-chain keto acids | short-chain keto acids | | ketoleucine/3-ketoisocaproic acid | - | 12 months before seroconversion | in GADA-first cases compared to controls (414 cases in a nested-case control study) | with autoantibody | Li Q, 2020 |
| | organic acids | keto acids | short-chain keto acids | short-chain keto acids | | ketoleucine/4-ketoisocaproic acid | + | at 3 months of age | 40 PT1D children compared to 40 P1Ab children and 40 matched controls | | Lamichhane S, 2019, Diabetologia |
| plasma sample | organic acids | keto acids | short-chain keto acids | short-chain keto acids | | alpha-ketoisovaleric acid | + | 9 months before seroconversion | in IAA-first cases compared to controls (414 cases in a nested-case control study) | with autoantibody | Li Q, 2020 |
| plasma sample | organic acids | keto acids | alpha-keto acids | alpha-keto acids | | pyruvic acid | + | at 3 months of age | 40 PT1D children compared to 40 P1Ab children and 40 matched controls | | Lamichhane S, 2019, Diabetologia |
| plasma sample | organic acids | keto acids | alpha-keto acids | alpha-keto acids | | pyruvic acid | - | at 27 months of age | in GADA-first cases compared to controls (414 cases in a nested-case control study) | with autoantibody | Li Q, 2020 |
| plasma sample | nucleosides, nucleotides, and analogues | pyrimidine nucleosides | | pyrimidine nucleosides | | uridine | + | at 9 months of age | in IAA-first cases compared to controls (414 cases in a nested-case control study) | with autoantibody | Li Q, 2020 |
| plasma sample | organoheterocyclic compounds | indoles and derivatives | indolyl carboxylic acids and derivatives | indolyl carboxylic acids and derivatives | | tryptophan | - | at 6 months of age | 20 PT1D children compared to 28 matched controls | | Lamichhane S, 2019, Diabetologia |
| serum sample | organoheterocyclic compounds | indoles and derivatives | indolyl carboxylic acids and derivatives | indolyl carboxylic acids and derivatives | | tryptophan | - | during 5-6yr | 56 PT1D children compared to 73 matched controls | | Oresic M, 2008 |
| cord blood sample | organoheterocyclic compounds | indoles and derivatives | indolyl carboxylic acids and derivatives | indolyl carboxylic acids and derivatives | | tryptophan | - | at birth | 15 PT1D children compared to 24 matched controls | | Oresic M, 2008 |
| plasma sample | organoheterocyclic compounds | indoles and derivatives | indolyl carboxylic acids and derivatives | indolyl carboxylic acids and derivatives | tryptophan derivative, microbial catabolites | indolelactic acid/indole-3-lactate | + | 3 months before seroconversion | in IAA-first cases compared to controls (414 cases in a nested-case control study) | with autoantibody | Li Q, 2020 |
| plasma sample | organoheterocyclic compounds | indoles and derivatives | tryptamines and derivatives | tryptamines and derivatives | | 5-methoxytrptamine | - | at 6 months of age | in IAA-first cases compared to controls (414 cases in a nested-case control study) | with autoantibody | Li Q, 2020 |
| plasma sample | organoheterocyclic compounds | indoles and derivatives | tryptamines and derivatives | tryptamines and derivatives | | 5-methoxytrptamine | + | at 9 months of age | in IAA-first cases compared to controls (414 cases in a nested-case control study) | with autoantibody | Li Q, 2020 |
| plasma sample | organoheterocyclic compounds | imidazopyrimidines | purines and purine derivatives | xanthines | | uric acid | + | at 3 months of age | in IAA-first cases compared to controls (414 cases in a nested-case control study) | with autoantibody | Li Q, 2020 |
| plasma sample | organoheterocyclic compounds | diazines | pyrimidines | pyrimidines | | uracil | + | at 24 months of age | in IAA-first cases compared to controls (414 cases in a nested-case control study) | with autoantibody | Li Q, 2020 |
| plasma sample | organoheterocyclic compounds | oxepanes | | oxepanes | oxepanes | levoglucosan | + | at 3 months of age | 40 PT1D children compared to 40 P1Ab children and 40 matched controls | | Lamichhane S, 2019, Diabetologia |
| plasma sample | organoheterocyclic compounds | oxepanes | | oxepanes | oxepanes | levoglucosan | + | at 15 months of age, 12 months before seroconversion | in IAA-first cases which experienced seroconversion after 2 years of age compared to controls (414 cases in a nested-case control study) | with autoantibody | Li Q, 2020 |
| plasma sample | organoheterocyclic compounds | lactones | gamma butyrolactones | gamma butyrolactones | | DHAA | + | at 3 months of age | in 414 IAA and GADA-first cases compared to controls (in a nested-case control study) | | Li Q, 2020 |
| plasma sample | organoheterocyclic compounds | lactams | caprolactams | caprolactams | | ε-caprolactam | - | at 6 months of age | in IAA-first cases compared to controls (414 cases in a nested-case control study) | with autoantibody | Li Q, 2020 |
| plasma sample | organoheterocyclic compounds | lactams | caprolactams | caprolactams | | ε-caprolactam | + | at 24 months of age | in IAA-first cases compared to controls (414 cases in a nested-case control study) | with autoantibody | Li Q, 2020 |
| plasma sample | organoheterocyclic compounds | piperidines | piperidinones | piperidinones | | piperidone | + | at 27,30,33,36 months of age | in GADA-first cases which experienced seroconversion after 2 years of age compared to controls (414 cases in a nested-case control study) | with autoantibody | Li Q, 2020 |
| plasma sample | organoheterocyclic compounds | piperidines | piperidinones | piperidinones | | piperidone | + | at 30 months of age | in GADA-first cases compared to controls (414 cases in a nested-case control study) | with autoantibody | Li Q, 2020 |
| serum sample | organoheterocyclic compounds | pyridines and derivatives | pyridinecarboxylic acids and derivatives | pyridinecarboxylic acids and derivatives | | alpha-ketoglutaric acid | - | at birth | 15 PT1D children compared to 24 matched controls | | Oresic M, 2008 |
| plasma sample | organoheterocyclic compounds | dihydrofurans | furanones | butenolides | vitamin C | ascorbic acid | - | at 6 months of age | 68 IAA-first case subjects (early IAA) compared to controls | | Li Q, 2021 |
| serum sample | organic nitrogen compounds | organonitrogen compounds | amines | monoalkylamines | | ethylamine | - | during 2-3yr | 56 PT1D children compared to 73 matched controls | | Oresic M, 2008 |
| plasma sample | organic nitrogen compounds | organonitrogen compounds | amines | 1,2-aminoalcohols | | ethanolamine | + | at 3 months of age | in GADA-first cases compared to controls (414 cases in a nested-case control study) | with autoantibody | Li Q, 2020 |
| plasma sample | organic nitrogen compounds | organonitrogen compounds | amines | 1,2-aminoalcohols | | ethanolamine | - | at 21 months of age | in GADA-first cases compared to controls (414 cases in a nested-case control study) | with autoantibody | Li Q, 2020 |
| serum sample | organic nitrogen compounds | organonitrogen compounds | amines | 1,2-aminoalcohols | | ethanolamine | - | at seroconversion | 13 autoantibody-positive children with ABs developed at or before 2 years old compared to matched controls | | Pflueger M, 2011 |
| plasma sample | organic nitrogen compounds | organonitrogen compounds | amines | N-acylethanolamines | | lactamide | + | at 6 months of age, 3 months before seroconversion | in IAA-first cases compared to controls (414 cases in a nested-case control study) | with autoantibody | Li Q, 2020 |
| dried blood spot sample | organic nitrogen compounds | organonitrogen compounds | quaternary ammonium salts | carnitines | *total carnitine, free carnitine, acyl-carnitine* | carnitine | - | at birth | 50 PT1D children compared to 200 matched controls | | la Marca G, 2013 |
| plasma sample | organic oxygen compounds | organooxygen compounds | carbohydrates | acylaminosugars | | N-acetylmannosamine | - | 12 months before seroconversion | in GADA-first cases compared to controls (414 cases in a nested-case control study) | with autoantibody | Li Q, 2020 |
| plasma sample | organic oxygen compounds | organooxygen compounds | carbohydrates | pentoses | | D-Arabinose | - | at 6 months of age | 20 PT1D children compared to 28 matched controls | | Lamichhane S, 2019, Diabetologia |
| plasma sample | organic oxygen compounds | organooxygen compounds | carbohydrates | pentoses | | ribose | + | at 24 months of age | in GADA-first cases compared to controls (414 cases in a nested-case control study) | with autoantibody | Li Q, 2020 |
| plasma sample | organic oxygen compounds | organooxygen compounds | carbohydrates | pentoses | | xylose | - | at 12 months of age, 3 months before seroconversion | in GADA-first cases compared to controls (414 cases in a nested-case control study) | with autoantibody | Li Q, 2020 |
| plasma sample | organic oxygen compounds | organooxygen compounds | carbohydrates | pentoses | | xylose | + | 3 months before seroconversion | in IAA-first cases compared to controls (414 cases in a nested-case control study) | with autoantibody | Li Q, 2020 |

| | | | | | | | | | | | |
|---|---|---|---|---|---|---|---|---|---|---|---|
| plasma sample | organic oxygen compounds | organooxygen compounds | carbohydrates | pentoses | | xylose | + | 12 months before seroconversion | in GADA-first cases compared to controls (414 cases in a nested-case control study) | with autoantibody | Li Q, 2020 |
| plasma sample | organic oxygen compounds | organooxygen compounds | carbohydrates | hexoses | | glucose | + | under poor glycemic control | 7 T1D children compared to 7 matched controls | | Bervoets L, 2017 |
| serum sample | organic oxygen compounds | organooxygen compounds | carbohydrates | hexoses | | D-galactofuranose | - | during 2-3yr | 56 PT1D children compared to 73 matched controls | | Oresic M, 2008 |
| serum sample | organic oxygen compounds | organooxygen compounds | carbohydrates | hexoses | | D-galactofuranose | + | during 4-5yr | 56 PT1D children compared to 73 matched controls | | Oresic M, 2008 |
| serum sample | organic oxygen compounds | organooxygen compounds | carbohydrates | hexoses | | glucopyranose/glucose | - | during 2-3yr | 56 PT1D children compared to 73 matched controls | | Oresic M, 2008 |
| serum sample | organic oxygen compounds | organooxygen compounds | carbohydrates | hexoses | | rhamnose | - | during 0-1yr | 56 PT1D children compared to 73 matched controls | | Oresic M, 2008 |
| PBMCs | organic oxygen compounds | organooxygen compounds | carbohydrates | hexose phosphates | | fructose-6-phosphate | - | at 12,36 months of age | 27 P1Ab children compared to 10 controls | | Sen P, 2020 |
| PBMCs | organic oxygen compounds | organooxygen compounds | carbohydrates | hexose phosphates | | fructose-6-phosphate | - | at 12,36 months of age | 34 PT1D children compared to 10 controls | | Sen P, 2020 |
| PBMCs | organic oxygen compounds | organooxygen compounds | carbohydrates | hexose phosphates | | glucose-6-phosphate | - | at 12,36 months of age | 27 P1Ab children compared to 10 controls | | Sen P, 2020 |
| PBMCs | organic oxygen compounds | organooxygen compounds | carbohydrates | hexose phosphates | | glucose-6-phosphate | + | at 24 months of age | 27 P1Ab children compared to 10 controls | | Sen P, 2020 |
| PBMCs | organic oxygen compounds | organooxygen compounds | carbohydrates | hexose phosphates | | glucose-6-phosphate | - | at 12,36 months of age | 34 PT1D children compared to 10 controls | | Sen P, 2020 |
| serum sample | organic oxygen compounds | organooxygen compounds | carbohydrates | glucuronic acid derivatives | | D-glucuronic acid | - | during 2-3yr | 56 PT1D children compared to 73 matched controls | | Oresic M, 2008 |
| serum sample | organic oxygen compounds | organooxygen compounds | carbohydrates | glucuronic acid derivatives | | D-glucuronic acid | + | during 4-5yr | 56 PT1D children compared to 73 matched controls | | Oresic M, 2008 |
| plasma sample | organic oxygen compounds | organooxygen compounds | carbohydrates | monosaccharides | glycolytic carbohydrates, anhydro sugar | 1,5-anhydroglucitol | - | at 6 months of age | 20 PT1D children compared to 28 matched controls | | Lamichhane S, 2019, Diabetologia |
| plasma sample | organic oxygen compounds | organooxygen compounds | carbohydrates | monosaccharides | | tagatose | - | 6 months before seroconversion | in IAA-first cases compared to controls (414 cases in a nested-case control study) | with autoantibody | Li Q, 2020 |
| PBMCs | organic oxygen compounds | organooxygen compounds | carbohydrates | monosaccharide phosphates | ketones, glycerone phosphates | dihydroxyacetone phosphate | - | at 12,36 months of age | 27 P1Ab children compared to 10 controls | | Sen P, 2020 |
| PBMCs | organic oxygen compounds | organooxygen compounds | carbohydrates | monosaccharide phosphates | ketones, glycerone phosphates | dihydroxyacetone phosphate | - | at 12 months of age | 34 PT1D children compared to 10 controls | | Sen P, 2020 |
| plasma sample | organic oxygen compounds | organooxygen compounds | carbohydrates | sugar acids | glyceric acid | glyceric acid | - | at 6 months of age | 20 PT1D children compared to 28 matched controls | | Lamichhane S, 2019, Diabetologia |
| plasma sample | organic oxygen compounds | organooxygen compounds | carbohydrates | sugar acids | threonic acid | threonic acid | - | at 6 months of age | 20 PT1D children compared to 28 matched controls | | Lamichhane S, 2019, Diabetologia |
| plasma sample | organic oxygen compounds | organooxygen compounds | carbohydrates | sugar acids | threonic acid | threonic acid | + | at 9 months of age | in IAA-first cases compared to controls (414 cases in a nested-case control study) | with autoantibody | Li Q, 2020 |
| plasma sample | organic oxygen compounds | organooxygen compounds | carbohydrates | sugar acids | ribonic acid | ribonic acid | - | at 6 months of age | 20 PT1D children compared to 28 matched controls | | Lamichhane S, 2019, Diabetologia |
| plasma sample | organic oxygen compounds | organooxygen compounds | carbohydrates | sugar acids | | 2,3-dihydroxybutanoic acid | - | at 9 months of age | in GADA-first cases compared to controls (414 cases in a nested-case control study) | with autoantibody | Li Q, 2020 |
| cord blood sample | organic oxygen compounds | organooxygen compounds | carbohydrates | sugar alcohols | | ribitol/xylitol | - | at birth | 15 PT1D children compared to 24 matched controls | | Oresic M, 2008 |
| plasma sample | organic oxygen compounds | organooxygen compounds | carbohydrates | sugar alcohols | | ribitol/xylitol | - | at 9 months of age | in GADA-first cases compared to controls (414 cases in a nested-case control study) | with autoantibody | Li Q, 2020 |
| plasma sample | organic oxygen compounds | organooxygen compounds | carbohydrates | sugar alcohols | | hexitol | - | 12 months before seroconversion | in GADA-first cases compared to controls (414 cases in a nested-case control study) | with autoantibody | Li Q, 2020 |
| plasma sample | organic oxygen compounds | organooxygen compounds | carbohydrates and carbohydrate conjugates | O-glycosyl compounds | | lactulose | - | at 3 months of age | in GADA-first cases compared to controls (414 cases in a nested-case control study) | with autoantibody | Li Q, 2020 |
| plasma sample | organic oxygen compounds | organooxygen compounds | carbohydrates and carbohydrate conjugates | O-glycosyl compounds | | lactose | - | at 18 months of age | in GADA-first cases compared to controls (414 cases in a nested-case control study) | with autoantibody | Li Q, 2020 |
| plasma sample | organic oxygen compounds | organooxygen compounds | carbonyl compounds | hydroxybenzaldehydes | | salicylaldehyde | - | 3 months before seroconversion | in GADA-first cases compared to controls (414 cases in a nested-case control study) | with autoantibody | Li Q, 2020 |
| plasma sample | organic oxygen compounds | organooxygen compounds | carbonyl compounds | hydroxybenzaldehydes | | salicylaldehyde | + | 6 and 9 months before seroconversion | in GADA-first cases compared to controls (414 cases in a nested-case control study) | with autoantibody | Li Q, 2020 |
| plasma sample | benzenoids | benzene and substituted derivatives | benzoic acids and derivatives | benzoic acids | | benzoic acid | - | at 18 months of age | in IAA-first cases compared to controls (414 cases in a nested-case control study) | with autoantibody | Li Q, 2020 |
| plasma sample | benzenoids | benzene and substituted derivatives | benzoic acids and derivatives | hippuric acids | | hippuric acid | - | at 21 months of age | in GADA-first cases compared to controls (414 cases in a nested-case control study) | with autoantibody | Li Q, 2020 |
| plasma sample | benzenoids | benzene and substituted derivatives | diphenylmethanes | bisphenols | | bisphenol A | + | at 3 months of age | 40 PT1D children compared to 40 P1Ab children and 40 matched controls | | Lamichhane S, 2019, Diabetologia |
| plasma sample | benzenoids | phenols | benzenediols | catechols | | 1,2-dihydroxycylohexane | + | 12 months before seroconversion | in IAA-first cases compared to controls (414 cases in a nested-case control study) | with autoantibody | Li Q, 2020 |
| plasma sample | phenylpropanoids and polyketides | benzene and substituted derivatives | | phenylpropanoic acids | phenols, microbial catabolites | 4-hydroxyphenyllactic acid | - | at 6 months of age | 20 PT1D children compared to 28 matched controls | | Lamichhane S, 2019, Diabetologia |
| plasma sample | homogeneous non-metal compounds | non-metal oxoanionic compounds | non-metal phosphates | non-metal phosphates | | phosphate | + | 9 months before seroconversion | in GADA-first cases compared to controls (414 cases in a nested-case control study) | with autoantibody | Li Q, 2020 |
| serum sample | homogeneous non-metal compounds | non-metal oxoanionic compounds | non-metal phosphates | non-metal phosphates | | phosphate | - | at birth | 13 PT1D children compared to 26 matched controls | | Oresic M, 2008 |
| serum sample | homogeneous non-metal compounds | non-metal oxoanionic compounds | non-metal phosphates | non-metal phosphates | | phosphate | - | during 3-4yr | 56 PT1D children compared to 73 matched controls | | Oresic M, 2008 |
| plasma sample | | | | | | *D-(-)-Lyxofuranose* | - | at 6 months of age | 20 PT1D children compared to 28 matched controls | | Lamichhane S, 2019, Diabetologia |

**Supplementary Table 2. Classified clusters of documented lipids and metabolites based on their chemical structures and physiological functions**

| No. | Lipid Cluster | Hits | Members |
|---|---|---|---|
| 1 | Unsaturated Triacylglycerols | 52 | TG(14:0/18:2/18:2), TG(16:0/16:0/18:1), TG(16:0/18:1/15:0), TG(16:0/18:1/16:0), TG(16:0/18:1/17:0), TG(16:0/18:1/18:0), TG(16:0/18:2/14:1), TG(16:0/18:2/18:2), TG(16:0/18:2/18:3), TG(16:0/18:3/18:2), TG(16:1/16:1/18:1), TG(17:0/18:1/18:2), TG(18:0/18:1/18:1), TG(18:1/14:0/16:0), TG(18:1/16:0/12:0), TG(18:1/18:1/15:0), TG(18:1/18:1/18:1), TG(18:1/18:2/18:2), TG(18:2/16:0/18:1), TG(18:2/18:1/18:1), TG(18:2/18:2/18:2), TG(46:1), TG(46:2), TG(47:1), TG(47:2), TG(48:1), TG(48:3), TG(49:3), TG(50:1), TG(50:2), TG(50:3), TG(50:4), TG(50:5), TG(51:1), TG(51:3), TG(51:4), TG(52:5), TG(53:2), TG(54:2), TG(54:3), TG(54:4), TG(54:5), TG(54:6), TG(55:2), TG(55:3),TG(55:4), TG(56:2), TG(56:6), TG(56:7), TG(56:8), TG(58:1), TG(58:8) |
| 2 | Unsatured Phosphatidylcholines | 29 | PC(14:0/18:2), PC(16:0/16:1), PC(16:0/18:1), PC(16:0/18:3), PC(16:0/20:5), PC(18:0/18:1), PC(18:0/20:3), PC(18:0/22:6), PC(18:2/16:1), PC(18:2/18:2), PC(18:2/20:4), PC(32:1), PC(32:2), PC(34:2), PC(34:3), PC(35:3), PC(36:2), PC(36:3), PC(36:4), PC(36:5), PC(37:3), PC(37:4), PC(38:3), PC(38:4), PC(38:5), PC(38:7), PC(40:4), PC(40:5), PC(40:7) |
| 3 | Sphingomyelins | 22 | SM(18:0/24:2), SM(d16:1/24:0), SM(d18:0/16:0), SM(d18:0/18:0), SM(d18:0/20:0), SM(d18:0/24:1), SM(d18:0/24:2), SM(d18:1/16:0), SM(d18:1/18:0), SM(d18:1/18:1), SM(d18:1/18:2), SM(d18:1/20:0), SM(d18:1/20:1), SM(d18:1/23:1), SM(d18:1/24:0), SM(d18:1/24:1), SM(d18:1/25:1), SM(d18:2/24:1), SM(d20:1/22:3), SM(d36:2), SM(d41:1), SM(d41:2) |
| 4 | Ether Phosphatidylcholines | 19 | PC(16:0e/18:2;16:0p/18:1), PC(16:0p/20:1;16:0e/20:2), PC(16:0p/22:2; 16:0e/22:3), PC(16:0p/22:4;16:0e/22:5), PC(18:0p/22:4;18:0e/22:5), PC(O-16:0/20:4), PC(O-18:1/20:4), PC(O-22:2/22:3), PC(O-32:0), PC(O-32:1), PC(O-34:1), PC(O-36:5), PC(O-38:4) or PC(P-38:3), PC(O-38:5), PC(O-40:4), PC(O-40:5), PC(P-16:0/16:0), PC(P-18:0/22:6), PC(P-32:0) |
| 5 | Lysophosphatidylcholines | 14 | LPC(14:0), LPC(16:0), LPC(16:1), LPC(18:0), LPC(18:1), LPC(18:2), LPC(18:3), LPC(20:0), LPC(20:1), LPC(20:2), LPC(20:3), LPC(20:5), LPC(22:0), LPC(22:6) |
| 6 | Oxidized Phosphatidylcholines | 7 | Oxidized PC(1-16:0/2-O-Hydroxy-7:2)/(16:0/7:1), Oxidized PC(16:0/Hydroxy-18:2), Oxidized PC(1-16:0/2-Hydroxy-18:3), Oxidized PC(16:0/Hydroperoxy-18:3), Oxidized PC(1-18:0/2-C7-oxo), Oxidized PC(18:0/Hydroxy-18:3), Oxidized PC(1-20:0/2-C7-oxo) |
| 7 | Phosphatidylethanolamines | 6 | PE(34:2), PE(36:2), PE(36:4), PE(38:5), PE(34:4e), PE(38:4e) |
| 8 | Lysophosphatidylethanolamines | 5 | LPE(16:0), LPE(18:2), LPE(22:6), LPE(O-18:1), LPE(P-16:0) |
| 9 | Satured Phosphatidylcholines | 5 | PC(17:0/2:0), PC(30:0), PC(32:0), PC (33:0), PC(34:0) |
| 10 | Cholesteryl Esters | 4 | CE(18:1), CE(18:2), CE(20:3), CE(20:5) |
| 11 | Ceramides | 4 | Cer(d18:1/22:0), Cer(d18:1/22:6), Cer(d18:1/23:0), Cer(d18:1/24:0) |
| 12 | Saturated Triacylglycerols | 4 | TG(16:0/18:0/16:0), TG(18:0/18:0/18:0), TG(42:0), TG(52:0) |
| 13 | Diradylglycerols | 2 | DG(33:5), DG(34:1) |
| 14 | Ether Phosphatidylethanolamines | 1 | PE(O-18:1(1Z)/20:4) |
| 15 | Glycosylceramides | 1 | GlcCer(d41:1) |
| 16 | Phosphatidylinositols | 1 | PI(18:0/20:4) |
| Total | | 176 | |

| No. | Metabolite Cluster | Hits | Members |
|---|---|---|---|
| 1 | Amino Acids | 22 | glycine, serine, alanine, N-methylalanine, tyrosine, methionine, phenylalanine, proline, L-hydroxyproline, aspartic acid, creatinine, glutamic acid, glutamine, ornithine, L-5-oxoproline, gamma-aminobutyric acid, cystine, 1-methylhistidine, tryptophan, lysine, threonine, L-cysteine |
| 2 | Monosaccharides | 12 | levoglucosan, N-acetylmannosamine, D-Arabinose, ribose, xylose, D-galactofuranose, glucose, rhamnose, D-glucuronic acid, 1,5-anhydroglucitol, tagatose, D-(-)-Lyxofuranose |
| 3 | Saturated Fatty Acids | 9 | lauric acid, pelargonic acid, palmitic acid, stearic acid, pentadecanoic acid, acetic acid, adipic acid, myristic acid, glutaric acid |
| 4 | Benzenoids | 8 | indolelactic acid, 5-methoxytryptamine, salicylaldehyde, benzoic acid, hippuric acid, bisphenol A, 1,2-dihydroxycyclohexane, 4-hydroxyphenyllactic acid |
| 5 | Unsaturated Fatty Acids | 7 | palmitoleic acid, oleic acid, arachidonic acid, 11-eicosenoic acid, adrenic acid, linoleic acid, linolenic acid |
| 6 | Phosphate Esters | 7 | glycerol-2-phosphate, methylphosphate, phosphoethanolamine, ethanolamine, phosphate, diglycerol, phosphoenolpyruvic acid |
| 7 | Hydroxy Fatty Acids | 6 | 2-hydroxy capric acid, 11,12-dihydroxy arachidic acid, 2-hydroxybutyric acid, 3-hydroxybutyric acid, 2-hydroxyglutaric acid, 2,4-dihydroxybutanoic acid |
| 8 | Lactams | 5 | uridine, uric acid, uracil, epsilon-caprolactam, piperidone |
| 9 | Sterols | 4 | campesterol, beta-sitosterol, cholesterol, lathosterol |
| 10 | Vitamins | 4 | 25-hydroxyvitamin D, alpha-tocopherol, dehydroascorbic acid, ascorbic acid |
| 11 | Sugar Acids | 4 | glyceric acid, threonic acid, ribonic acid, 2,3-dihydroxybutanoic acid |
| 12 | TCA Cycle | 4 | succinic acid, malic acid, citric acid, alpha-ketoglutaric acid |
| 13 | Branched-Chain Amino Acids | 3 | valine, leucine, isoleucine |
| 14 | Sugar Phosphates | 3 | dihydroxyacetone phosphate, fructose-6-phosphate, glucose-6-phosphate |
| 15 | Keto Acids | 3 | ketoleucine, alpha-ketoisovaleric acid, pyruvic acid |
| 16 | Monoradylglycerols | 2 | 1-monopalmitin, 1-monoolein |
| 17 | Bile Acids | 2 | deoxycholic acid 3-glucuronide, 3,7,12-trioxochola-1,4-dien-24-oic acid |
| 18 | Fatty Alcohols | 2 | 1-dodecanol, octadecanol |
| 19 | Sugar Alcohols | 2 | ribitol, hexitol |
| 20 | Disaccharides | 2 | lactulose, lactose |
| 21 | Amines | 2 | ethylamine, carnitine |
| 22 | Amides | 1 | lactamide |
| 23 | Branched Fatty Acids | 1 | itaconic acid |
| 24 | Steroids | 1 | tetrahydroaldosterone-3-glucuronide |
| 25 | Hydroxy Acids | 1 | lactic acid |
| Total | | 117 | |

Supplementary Table 3. Labeling clusters according to the tentative effective-score rule

| No. | Cluster | Hits | at birth | 3m | 6m | 9m | 1yr | 1-2yr | 2yr | 2-3yr | 3yr | 3-4yr | 4-10yr | -1yr | -6m | -3m | B-Ab+ | Ab+ | +3m | +6m | +1yr | B-T1D | T1D | poor | good |
|---|---|---|---|---|---|---|---|---|---|---|---|---|---|---|---|---|---|---|---|---|---|---|---|---|---|
| 1 | Unsaturated Triacylglycerols | 52 | -6 | | -10 | | -30 | -12 | 3 | -7 | -13 | -6 | -9 | | | | -1 | 5 | | | 3 | | -3 | | |
| 2 | Unsatured Phosphatidylcholines | 29 | -4 | 9 | -1 | -1 | a | | 7 | | -12 | | -17 | | | -1 | | 4 | | | 3 | | e | | |
| 3 | Amino Acids | 22 | -6 | -2 | -6 | -1 | -9 | -2 | 11 | -4 | -7 | | -2 | a | -2 | 1 | | -7 | -3 | | -2 | | | -2 | |
| 4 | Sphingomyelins | 22 | -11 | -1 | -2 | -1 | a | -3 | 1 | | -4 | | -4 | | | | | | | | | | -9 | | |
| 5 | Ether Phosphatidylcholines | 19 | a | | | -2 | -1 | -3 | 7 | -1 | -6 | | -3 | -3 | | -1 | | | -4 | | -4 | -4 | -3 | | |
| 6 | Lysophosphatidylcholines | 14 | 1 | | 1 | | -4 | 1 | 3 | | 1 | | 1 | a | f | 1 | | 1 | -2 | | -1 | -1 | 2 | -6 | -1 |
| 7 | Monosaccharides | 12 | | 1 | -4 | | -1 | 1 | 1 | -3 | | 1 | 1 | 1 | -1 | a | | | | | | | | 1 | |
| 8 | Saturated Fatty Acids | 9 | | -3 | -1 | a | -2 | | 1 | -1 | a | -1 | | | | | | 1 | | | | | | | 1 |
| 9 | Benzenoids | 8 | | 1 | -2 | 1 | | -2 | | | | | | 1 | 2 | a | | | | | | | | | |
| 10 | Phosphate Esters | 7 | -1 | b | 1 | | -2 | -1 | | | -2 | -1 | | 1 | 1 | | | -1 | | | | | | | |
| 11 | Unsaturated Fatty Acids | 7 | | -4 | | | | 1 | | -1 | | | 1 | | | 1 | | | | | | | | | 4 |
| 12 | Oxidized Phosphatidylcholines | 7 | | | | | | | | | | | | | | | | | | | | | 7 | | |
| 13 | Hydroxy Fatty Acids | 6 | | | | 1 | | | -1 | | | | | | | -1 | | -1 | 1 | | 1 | | | | a |
| 14 | Phosphatidylethanolamines | 6 | -1 | 2 | | -1 | | | | | | | | | | | -1 | a | | | 1 | | a | | |
| 15 | Lactams | 5 | | 1 | -1 | 1 | | | 2 | 3 | 1 | | | | | | | | | | | | | | |
| 16 | Lysophosphatidylethanolamines | 5 | | | | | | | | | | | | | | | | | | | | | 2 | | 3 |
| 17 | Satured Phosphatidylcholines | 5 | | | | | -1 | -1 | 1 | | -1 | | | | | | | | | | | | 1 | | 1 |
| 18 | Sterols | 4 | | | | -1 | | | | | | -1 | | | | | | | | | | | | 1 | |
| 19 | Sugar Acids | 4 | | | -3 | a | | | | | | | | | | | | | | | | | | | |
| 20 | TCA Cycle | 4 | -3 | | -2 | 2 | -1 | | -1 | -1 | -2 | -1 | | -1 | | 1 | | | -1 | | -1 | | | | |
| 21 | Vitamins | 4 | | 1 | -2 | | | | | | | | | | 1 | | -1 | | | | | | | | |
| 22 | Cholesteryl Esters | 4 | 1 | | | | -1 | | 1 | | -1 | | | | | | | | | | | | a | | |
| 23 | Ceramides | 4 | | | -1 | | | | 1 | | -6 | | | | | | | 1 | | | | | | | |
| 24 | Saturated Triacylglycerols | 4 | | | | | 1 | | 1 | -2 | -4 | | | | | | | | | | | | | | |
| 25 | Branched-Chain Amino Acids | 3 | | | | | 3 | -6 | -1 | | a | | | 2 | | 1 | 1 | -2 | | | | | | | |
| 26 | Keto Acids | 3 | | 2 | | | | | | -1 | | | | -2 | 1 | -1 | | | | | | | | | |
| 27 | Sugar Phosphates | 3 | | | | | -6 | | 1 | | -5 | | | | | | | | | | | | | | |
| 28 | Amines | 2 | -1 | | | | | | | -1 | | | | | | | | | | | | | | | |
| 29 | Bile Acids | 2 | | | | | | | | | | | | | | | | | | | | | | | a |
| 30 | Disaccharides | 2 | | -1 | | | | -1 | | | | | | | | | | | | | | | | | |
| 31 | Fatty Alcohols | 2 | | | | | | | | 1 | 1 | | | | | | | | | | | | | | |
| 32 | Monoradylglycerols | 2 | | -1 | | 1 | | | | | | | | 1 | | | | | | | | | | | |
| 33 | Sugar Alcohols | 2 | -1 | | | -1 | | | | | | | | -1 | | | | | | | | | | | |
| 34 | Diradylglycerols | 2 | | | | | | | | | | | | | -1 | | | a | | 1 | | | | | |
| 35 | Amides | 1 | | | 1 | | | | | | | | | | | 1 | | | | | | | | | |
| 36 | Branched Fatty Acids | 1 | | | 1 | | | | | | | | | | | | | | | | | | | | |
| 37 | Hydroxy Acids | 1 | -1 | | | | 1 | | | | | | | | | | | | 1 | | | | | | |
| 38 | Steroids | 1 | | | | | | | | | | | | | | | | | | | | | | | -1 |
| 39 | Ether Phosphatidylethanolamines | 1 | | | | | -1 | | | | | | | | | | | | | | | | | | |
| 40 | Glycosylceramides | 1 | | | | -1 | | | | | | | | | | | | | | | | | | | |
| 41 | Phosphatidylinositols | 1 | | | | | | | 1 | | -2 | | | | | | | | | | | | | | |

Supplementary Table 4. Metabolite profiling throughout the occurrence and progress of T1D based on the quantitative effect-score rule

| Compound Name | Cluster | PubChem | HMDB ID | at birth | 3m | 6m | 9m | 1yr | 15m | 18m | 21m | 2yr | 27m | 30m | 33m | 3yr | 3-4yr | 4-5yr | 5-6yr | 6-7yr | 7-8yr | 8-9yr | 9-10yr | -18m | -13.5m | -12m | -9m | -6m | -4.5m | -3m | B-Ab+ | Ab+ | A-Ab+ | +3m | +4.5m | +6m | +1yr | +13.5m | -31.5m | -22.5m | T1D | treatment | poor | good |
|---|---|---|---|---|---|---|---|---|---|---|---|---|---|---|---|---|---|---|---|---|---|---|---|---|---|---|---|---|---|---|---|---|---|---|---|---|---|---|---|---|---|---|---|---|
| glycerol-2-phosphate | Phosphate Esters | 2526 | HMDB0002520 | | 1 | | | | | | | | | | | | | | | | | | | | | | | | | | | | | | | | | | | | | | | |
| 1-monopalmitin | Monoradylglycerols | 14900 | HMDB0031074 | | -1 | | | | | | | | | | | | | | | | | | | | | | | | | | | | | | | | | | | | | | | |
| 1-monoolein | Monoradylglycerols | 5283468 | HMDB0094684 | | | | 1 | | | | | | | | | | | | | | | | | | | 1 | | | | | | | | | | | | | | | | | | |
| deoxycholic acid 3-glucuronide | Bile Acids | 44263368 | HMDB0002596 | | | | | | | | | | | | | | | | | | | | | | | | | | | | | | | | | | | | | | | | | 1 |
| tetrahydroaldosterone-3-glucuronide | Steroids | 167918 | HMDB0010357 | | | | | | | | | | | | | | | | | | | | | | | | | | | | | | | | | | | | | | | | | -1 |
| 3,7,12-trioxochola-1,4-dien-24-oic acid | Bile Acids | 5284110 | | | | | | | | | | | | | | | | | | | | | | | | | | | | | | | | | | | | | | | | | | -1 |
| campesterol | Sterols | 173183 | HMDB0002869 | | | | | | | | | | | | | | | | | | | | | | | | | | | | | | | | | | | | | | | | 1 | |
| beta-sitosterol | Sterols | 222284 | HMDB0000852 | | | | | | | | | | | | | | | | | | | | | | | | | | | | | | | | | | | | | | | | 1 | |
| cholesterol | Sterols | 5997 | HMDB0000067 | | | | -1 | | | | | | | | | | -1 | | | | | | | | | | | | | | | | | | | | | | | | | | | |
| lathosterol | Sterols | 65728 | HMDB0001170 | | | | | | | | | | | | | | | | | | | | | | | | | | | | | | | | | | | | | | | | -1 | |
| 25-hydroxyvitamin D | Vitamins | 5283731 | HMDB0003550 | | | -1 | | | | | | | | | | | | | | | | | | | | | | | | | -1 | | | | | | | | | | | | | |
| lauric acid | Saturated Fatty Acids | 3893 | HMDB0000638 | | -1 | | | | | | | | | -1 | | | | | | | | | | | | | | | | | | | | | | | | | | | | | | |
| pelargonic acid | Saturated Fatty Acids | 8158 | HMDB0000847 | | | | -1 | | | | | | | | | | | | | | | | | | | | | | | | | | | | | | | | | | | | | |
| 2-hydroxy capric acid | Hydroxy Fatty Acids | 21488 | HMDB0094656 | | | | | | | | | | | | | | | | | | | | | | | | | | | | | | | | | | | | | | | | | -1 |
| 11,12-dihydroxy arachidic acid | Hydroxy Fatty Acids | 5282932 | | | | | | | | | | | | | | | | | | | | | | | | | | | | | | | | | | | | | | | | | | 1 |
| palmitic acid | Saturated Fatty Acids | 985 | HMDB0000220 | | -1 | | | | | | | | | | | 1 | | | | | | | | | | | | | | | | | | | | | | | | | | | | 1 |
| palmitoleic acid | Unsaturated Fatty Acids | 445638 | HMDB0003229 | | | | | | | | | | | | | | | 1 | | | | | | | | | | | | 1 | | | | | | | | | | | | | | 1 |
| oleic acid | Unsaturated Fatty Acids | 445639 | HMDB0000207 | | -1 | | | | | | | | | | | | | | | | | | | | | | | | | | | | | | | | | | | | | | | 1 |
| stearic acid | Saturated Fatty Acids | 5281 | HMDB0000827 | | -1 | | | | | | | | | | | | | | | | | | | | | | | | | | | | | | | | | | | | | | | |
| arachidonic acid | Unsaturated Fatty Acids | 444899 | HMDB0001043 | | -1 | | | | 1 | | | | | | | | | | | | | | | | | | | | | | | | | | | | | | | | | | | |
| pentadecanoic acid | Saturated Fatty Acids | 13849 | HMDB0000826 | | | -1 | | | | | | | | | | | | | | | | | | | | | | | | | | | | | | | | | | | | | | |
| 11-eicosenoic acid | Unsaturated Fatty Acids | 5282768 | HMDB0002231 | | -1 | | | | | | | | | | | | | | | | | | | | | | | | | | | | | | | | | | | | | | | |
| adrenic acid | Unsaturated Fatty Acids | 5497181 | HMDB0002226 | | | | | | | | | | | | | | | | | | | | | | | | | | | | | | | | | | | | | | | | | 1 |
| 1-dodecanol | Fatty Alcohols | 8193 | HMDB0011626 | | | | | | | | | | | | | 1 | | | | | | | | | | | | | | | | | | | | | | | | | | | | |
| octadecanol | Fatty Alcohols | 8221 | HMDB0002350 | | | | | | | | | | | | 1 | | | | | | | | | | | | | | | | | | | | | | | | | | | | | |
| linoleic acid | Unsaturated Fatty Acids | 5280450 | HMDB0000673 | | -1 | | | | | | | | | -1 | | | | | | | | | | | | | | | | | | | | | | | | | | | | | | |
| linolenic acid | Unsaturated Fatty Acids | 5280934 | HMDB0001388 | | | | | | | | | | | | | | | | | | | | | | | | | | | | | | | | | | | | | | | | | 1 |
| alpha-tocopherol | Vitamins | 14985 | HMDB0001893 | | | | | | | | | | | | | | | | | | | | | | | | | 1 | | | | | | | | | | | | | | | | |
| acetic acid | Saturated Fatty Acids | 176 | HMDB0000042 | | | | | | | | | | | | | | -1 | | | | | | | | | | | | | | | | | | | | | | | | | | | |
| adipic acid | Saturated Fatty Acids | 196 | HMDB0000448 | | | | 1 | | | | | | | | | | | | | | | | | | | | | | | | | | | | | | | | | | | | | |
| myristic acid | Saturated Fatty Acids | 11005 | HMDB0000806 | | | | | -2 | | | | | | | | | | | | | | | | | | | | | | | | | | | | | | | | | | | | |
| glutaric acid | Saturated Fatty Acids | 743 | HMDB0000661 | | | | | | | | | 1 | | | | -1 | | | | | | | | | | | | | | | | | | | | | | | | | | | | |
| succinic acid | TCA Cycle | 1110 | HMDB0000254 | -1 | | -2 | 1 | | | | | | | | | | -1 | | | | | | | | | | | | 1 | | | | | | | | | | | | | | | |
| malic acid | TCA Cycle | 525 | HMDB0000156 | | | | 1 | | | | | | | | | | | | | | | | | | | | | | | | | | | | | | | | | | | | | |
| citric acid | TCA Cycle | 311 | HMDB0000094 | -1 | | | | -1 | | | | -1 | | -1 | | -2 | | | | | | | | | -1 | | | | | | | | | -1 | | | | -1 | | | | | | |
| itaconic acid | Branched Fatty Acids | 811 | HMDB0002092 | | | 1 | | | | | | | | | | | | | | | | | | | | | | | | | | | | | | | | | | | | | | |
| valine | Branched-Chain Amino Acids | 6287 | HMDB0000883 | | | | | 2 | -1 | -1 | -1 | -1 | | | | | | | | | | | | | | | | | | | | -1 | | | | | | | | | | | | |
| leucine | Branched-Chain Amino Acids | 6106 | HMDB0000687 | | | | | | | | | | | | | | | | | | | | | | 1 | | | | 1 | | | -1 | | | | | | | | | | | | |
| isoleucine | Branched-Chain Amino Acids | 6306 | HMDB0000172 | | | | | | -1 | -1 | -1 | -1 | | | | | | | | | | | | | 1 | | | | | | 1 | | | | | | | | | | | | | |
| glycine | Amino Acids | 750 | HMDB0000123 | -1 | | | | | | | | | | | | | | | | | | | | | | | | | | | | | | | | | | | | | | | | |
| serine | Amino Acids | 5951 | HMDB0000187 | -1 | | | | | | | | 2 | | | | -1 | | | | | | | | | | | | | | | | | | | | | | | | | | | -1 | |
| alanine | Amino Acids | 602 | HMDB0062251 | -1 | | | | -1 | | | | 2 | | | | 2 | | | -1 | | | | | | | -1 | | -1 | a | | | | | | | | | | | | | | | |
| N-methylalanine | Amino Acids | 5288725 | HMDB0094692 | | | | | | | | | | | | | | | | | | | | | | | | | -1 | | | | | | | | | | | | | | | | |
| tyrosine | Amino Acids | 6057 | HMDB0000158 | | | | | -1 | | | | | | | | | | | | | | | | | | | | | | | | -1 | | -1 | | | | | | | | | | |
| methionine | Amino Acids | 876 | HMDB0033951 | | 1 | a | | | | | | | | | | | | | | | | | | | | | | | | | | -1 | | | | | -1 | | | | | | | |
| phenylalanine | Amino Acids | 6140 | HMDB0000159 | | -1 | | | -1 | | | | 2 | | | | -2 | | | | | | | | | | | | | | | | | | | | | | | | | | | | |
| proline | Amino Acids | 145742 | HMDB0000162 | | -1 | -1 | -1 | -1 | | -1 | -1 | 1 | -1 | -1 | -1 | -1 | | | | | | | | | | | | | | | | | | | | | | | | | | | | |
| L-hydroxyproline | Amino Acids | 5810 | HMDB0000725 | | | -1 | | | | | | | -1 | | | | | | | | | | | | | | | | | | | -1 | | | | | | | | | | | | |
| aspartic acid | Amino Acids | 5960 | HMDB0000191 | | | -1 | | | | | | 2 | | | | | | | | | | | | | | | | | | | | | | | | | | -1 | | | | | | |
| creatinine | Amino Acids | 588 | HMDB0000562 | -1 | | | | | | | | | | | | | | | | | | | | | | | | | | | | | | | | | | | | | | | | |
| glutamic acid | Amino Acids | 33032 | HMDB0000148 | | -1 | -1 | | | | | | 2 | | | | | | | | | | | | | 1 | | | | 1 | | | -1 | | | | | | | | | | | | |
| glutamine | Amino Acids | 5961 | HMDB0000641 | | | | | | | | | | | | | | | | | | | | | | -1 | | | | | | | | | | -1 | | | | | | | | | |
| ornithine | Amino Acids | 6262 | HMDB0000214 | -1 | | | | | | | | 1 | | | | | | | | | | | | | | | | | | | | -1 | | -1 | | | | | | | | | | |
| L-5-oxoproline | Amino Acids | 7405 | HMDB0000267 | | -1 | -1 | | | | | | -1 | | | | -2 | | | | | | | | | | | | | | | | | | | | | | | | | | | | |
| gamma-aminobutyric acid | Amino Acids | 119 | HMDB0000112 | | 1 | | | | | | | | | | | | | | | | | | | | 1 | | | | | | | | | | | | | | | | | | | |
| cystine | Amino Acids | 595 | HMDB0000192 | | | | | -2 | | | | | | | | -2 | | | | | | | | | | | | | | | | | | | | | | | | | | | | |
| 1-methylhistidine | Amino Acids | 92105 | HMDB0000001 | | | | | | | | | | | | | | | | | | | | | | | | | | | | | -1 | | | | | | | | | | | | |
| lysine | Amino Acids | 5962 | HMDB0000182 | | | | | -2 | | | | | | | | | | | | | | | | | | | | | | | | | | | | | | | | | | | | |
| threonine | Amino Acids | 6288 | HMDB0000167 | | | | | -1 | | | | | | | | -1 | | | | | | | | | | | | | | | | | | | | | | | | | | | | |
| L-cysteine | Amino Acids | 5862 | HMDB0000574 | | | | | | | | | | | | | | | | | | | | | | | | | | | | | | | | | | | | | | | | -1 | |
| lactic acid | Hydroxy Acids | 612 | HMDB0000190 | -1 | | | | 1 | | | | | | | | | | | | | | | | | | | | | | | | | | | 1 | | | | | | | | | |
| 2-hydroxybutyric acid | Hydroxy Fatty Acids | 11266 | HMDB0000008 | | | | | | | | | | | | | | | | | | | | | | | | | | -1 | | | | | | 1 | | | 1 | | | | | | |
| 3-hydroxybutyric acid | Hydroxy Fatty Acids | 441 | HMDB0000357 | | | | | | | | | -1 | | | | | | | | | | | | | | | | | | | | | | | | | | | | | | | | |
| 2-hydroxyglutaric acid | Hydroxy Fatty Acids | 43 | HMDB0059655 | | | | 1 | | | | | | | | | | | | | | | | | | | | | | | | | | | | | | | | | | | | | |
| 2,4-dihydroxybutanoic acid | Hydroxy Fatty Acids | 192742 | HMDB0000360 | | | | | | | | | | | | | | | | | | | | | | | | | | | | | -1 | | | | | | | | | | | | |
| methylphosphate | Phosphate Esters | 13130 | HMDB0061711 | | -1 | | | | | | | | | | | | | | | | | | | | | | | | | | | | | | | | | | | | | | | |
| phosphoethanolamine | Phosphate Esters | 1015 | HMDB0000224 | | | | | | | | | | | | | | | | | | | | | | | 1 | | | | | | | | | | | | | | | | | | |
| phosphoenolpyruvic acid | Phosphate Esters | 1005 | HMDB0000263 | | | | | -2 | | | | | | | | -2 | | | | | | | | | | | | | | | | | | | | | | | | | | | | |
| ketoleucine | Keto Acids | 70 | HMDB0000695 | | 1 | | | | | | | | | | | | | | | | | | | | -1 | -1 | | | -1 | | | | | | | | | | | | | | | |
| alpha-ketoisovaleric acid | Keto Acids | 49 | HMDB0000019 | | | | | | | | | | | | | | | | | | | | | | | | 1 | | | | | | | | | | | | | | | | | |
| pyruvic acid | Keto Acids | 1060 | HMDB0000243 | | 1 | | | | | | | | -1 | | | | | | | | | | | | | | | | | | | | | | | | | | | | | | | |
| uridine | Lactams | 6029 | HMDB0000296 | | | | 1 | | | | | | | | | | | | | | | | | | | | | | | | | | | | | | | | | | | | | |
| tryptophan | Amino Acids | 6305 | HMDB0000929 | -1 | | -1 | | | | | | | | | | | | | -1 | | | | | | | | | | | | | | | | | | | | | | | | | |
| indolelactic acid | Benzenoids | 92904 | HMDB0000671 | | | | | | | | | | | | | | | | | | | | | | | | | | | 1 | | | | | | | | | | | | | | |
| 5-methoxytryptamine | Benzenoids | 1833 | HMDB0004095 | | | -1 | 1 | | | | | | | | | | | | | | | | | | | | | | | | | | | | | | | | | | | | | |
| uric acid | Lactams | 1175 | HMDB0000289 | | 1 | | | | | | | | | | | | | | | | | | | | | | | | | | | | | | | | | | | | | | | |
| uracil | Lactams | 1174 | HMDB0000300 | | | | | | | | | 1 | | | | | | | | | | | | | | | | | | | | | | | | | | | | | | | | |
| levoglucosan | Monosaccharides | 2724705 | HMDB0000640 | | 1 | | | | 1 | | | | | | | | | | | | | | | | | 1 | | | | | | | | | | | | | | | | | | |
| dehydroascorbic acid | Vitamins | 440667 | HMDB0001264 | | 1 | | | | | | | | | | | | | | | | | | | | | | | | | | | | | | | | | | | | | | | |
| epsilon-caprolactam | Lactams | 7768 | HMDB0062769 | | | -1 | | | | | | 1 | | | | | | | | | | | | | | | | | | | | | | | | | | | | | | | | |
| piperidone | Lactams | 12665 | HMDB0011749 | | | | | | | | | | 1 | 1 | 1 | 1 | | | | | | | | | | | | | | | | | | | | | | | | | | | | |
| alpha-ketoglutaric acid | TCA Cycle | 51 | HMDB0000208 | -1 | | | | | | | | | | | | | | | | | | | | | | | | | | | | | | | | | | | | | | | | |
| ascorbic acid | Vitamins | 54670067 | HMDB0000044 | | | -1 | | | | | | | | | | | | | | | | | | | | | | | | | | | | | | | | | | | | | | |
| ethylamine | Amines | 6341 | HMDB0013231 | | | | | | | | | | | -1 | | | | | | | | | | | | | | | | | | | | | | | | | | | | | | |
| ethanolamine | Phosphate Esters | 700 | HMDB0000149 | | 1 | | | | | | -1 | | | | | | | | | | | | | | | | | | | | | -1 | | | | | | | | | | | | |
| lactamide | Amides | 94220 | | | | 1 | | | | | | | | | | | | | | | | | | | | | | | | 1 | | | | | | | | | | | | | | |
| carnitine | Amines | 288 | HMDB0000062 | -1 | | | | | | | | | | | | | | | | | | | | | | | | | | | | | | | | | | | | | | | | |
| N-acetylmannosamine | Monosaccharides | 11096158 | HMDB0001129 | | | | | | | | | | | | | | | | | | | | | | | -1 | | | | | | | | | | | | | | | | | | |
| D-Arabinose | Monosaccharides | 66308 | HMDB0029942 | | | -1 | | | | | | | | | | | | | | | | | | | | | | | | | | | | | | | | | | | | | | |
| ribose | Monosaccharides | 10975657 | HMDB0000283 | | | | | | | | | 1 | | | | | | | | | | | | | | | | | | | | | | | | | | | | | | | | |
| xylose | Monosaccharides | 135191 | HMDB0000098 | | | | | -1 | | | | | | | | | | | | | | | | | | 1 | | | | a | | | | | | | | | | | | | | |
| D-galactofuranose | Monosaccharides | 15560222 | | | | | | | | | | | | -1 | | | 1 | | | | | | | | | | | | | | | | | | | | | | | | | | | |
| glucose | Monosaccharides | 5793 | HMDB0000122 | | | | | | | | | | | -1 | | | | | | | | | | | | | | | | | | | | | | | | | | | | | 1 | |
| rhamnose | Monosaccharides | 25310 | HMDB0000174 | | | -1 | | | | | | | | | | | | | | | | | | | | | | | | | | | | | | | | | | | | | | |
| D-glucuronic acid | Monosaccharides | 94715 | HMDB0000127 | | | | | | | | | | | -1 | | | | 1 | | | | | | | | | | | | | | | | | | | | | | | | | | |
| 1,5-anhydroglucitol | Monosaccharides | 64960 | HMDB0002712 | | | -1 | | | | | | | | | | | | | | | | | | | | | | | | | | | | | | | | | | | | | | |
| tagatose | Monosaccharides | 439312 | HMDB0003418 | | | | | | | | | | | | | | | | | | | | | | | | | -1 | | | | | | | | | | | | | | | | |
| glyceric acid | Sugar Acids | 752 | HMDB0000139 | | | -1 | | | | | | | | | | | | | | | | | | | | | | | | | | | | | | | | | | | | | | |
| threonic acid | Sugar Acids | 5460407 | HMDB0000943 | | | -1 | 1 | | | | | | | | | | | | | | | | | | | | | | | | | | | | | | | | | | | | | |

| | | | | | | | | | | | | | | | | | | | | | | | | | | | | | | |
|---|---|---|---|---|---|---|---|---|---|---|---|---|---|---|---|---|---|---|---|---|---|---|---|---|---|---|---|---|---|---|
| ribonic acid | Sugar Acids | 5460677 | HMDB0000867 | | | -1 | | | | | | | | | | | | | | | | | | | | | | | | |
| 2,3-dihydroxybutanoic acid | Sugar Acids | 250402 | HMDB0000498 | | | | -1 | | | | | | | | | | | | | | | | | | | | | | | |
| ribitol | Sugar Alcohols | 6912 | HMDB0002917 | -1 | | | -1 | | | | | | | | | | | | | | | | | | | | | | | |
| hexitol | Sugar Alcohols | 453 | HMDB0000107 | | | | | | | | | | | | | | | | | | | | | | | -1 | | | | |
| dihydroxyacetone phosphate | Sugar Phosphates | 668 | HMDB0001473 | | | | | -2 | | | | | | | | -1 | | | | | | | | | | | | | | |
| fructose-6-phosphate | Sugar Phosphates | 69507 | HMDB0000124 | | | | | -2 | | | | | | | | -2 | | | | | | | | | | | | | | |
| glucose-6-phosphate | Sugar Phosphates | 5958 | HMDB0001401 | | | | | -2 | | | | 1 | | | | -2 | | | | | | | | | | | | | | |
| lactulose | Disaccharides | 11333 | HMDB0000740 | | -1 | | | | | | | | | | | | | | | | | | | | | | | | | |
| lactose | Disaccharides | 6134 | HMDB0041627 | | | | | | | -1 | | | | | | | | | | | | | | | | | | | | |
| salicylaldehyde | Benzenoids | 6998 | HMDB0034170 | | | | | | | | | | | | | | | | | | | | | | | | 1 | 1 | | -1 |
| benzoic acid | Benzenoids | 243 | HMDB0001870 | | | | | | | -1 | | | | | | | | | | | | | | | | | | | | |
| hippuric acid | Benzenoids | 464 | HMDB0000714 | | | | | | | | -1 | | | | | | | | | | | | | | | | | | | |
| bisphenol A | Benzenoids | 6623 | HMDB0032133 | | 1 | | | | | | | | | | | | | | | | | | | | | | | | | |
| 1,2-dihydroxycyclohexane | Benzenoids | 13601 | HMDB0000957 | | | | | | | | | | | | | | | | | | | | | | | 1 | | | | |
| 4-hydroxyphenyllactic acid | Benzenoids | 9378 | HMDB0000755 | | | -1 | | | | | | | | | | | | | | | | | | | | | | | | |
| phosphate | Phosphate Esters | 1061 | HMDB0001429 | -1 | | | | | | | | | | | | | -1 | | | | | | | | | | 1 | | | |
| D-(-)-Lyxofuranose | Monosaccharides | 11435035 | | | | -1 | | | | | | | | | | | | | | | | | | | | | | | | |
| diglycerol | Phosphate Esters | 42953 | HMDB0240316 | | -1 | 1 | | | | | | | | | | | | | | | | | | | | | | | | |

**Supplementary Table 5. Pathway analysis of involved metabolites and lipids based on the MetaboAnalyst platform**

| Metablic pathway | Total | Expected | Hits | Raw p | Holm p | FDR |
|---|---|---|---|---|---|---|
| Aminoacyl-tRNA biosynthesis | 48 | 1.89 | 16 | 3.64E-12 | 2.87E-10 | 2.87E-10 |
| Valine, leucine and isoleucine biosynthesis | 8 | 0.315 | 6 | 7.62E-08 | 5.94E-06 | 3.01E-06 |
| Alanine, aspartate and glutamate metabolism | 28 | 1.1 | 9 | 4.74E-07 | 3.65E-05 | 1.25E-05 |
| Arginine biosynthesis | 14 | 0.551 | 5 | 0.000122 | 0.00924 | 0.0024 |
| Glyoxylate and dicarboxylate metabolism | 32 | 1.26 | 7 | 0.000159 | 0.012 | 0.00252 |
| Glycolysis / Gluconeogenesis | 26 | 1.02 | 6 | 0.000359 | 0.0266 | 0.00473 |
| Pantothenate and CoA biosynthesis | 19 | 0.748 | 5 | 0.000606 | 0.0443 | 0.00684 |
| Citrate cycle (TCA cycle) | 20 | 0.787 | 5 | 0.000784 | 0.0565 | 0.00775 |
| D-Glutamine and D-glutamate metabolism | 6 | 0.236 | 3 | 0.00107 | 0.0756 | 0.00935 |
| Butanoate metabolism | 15 | 0.59 | 4 | 0.00214 | 0.15 | 0.016 |
| Biosynthesis of unsaturated fatty acids | 36 | 1.42 | 6 | 0.00223 | 0.154 | 0.016 |
| Arginine and proline metabolism | 38 | 1.5 | 6 | 0.00297 | 0.202 | 0.0196 |
| Glutathione metabolism | 28 | 1.1 | 5 | 0.0039 | 0.261 | 0.0237 |
| Phenylalanine metabolism | 10 | 0.393 | 3 | 0.00571 | 0.377 | 0.0322 |
| Glycine, serine and threonine metabolism | 33 | 1.3 | 5 | 0.00809 | 0.526 | 0.0426 |
| Pyruvate metabolism | 22 | 0.866 | 4 | 0.00931 | 0.596 | 0.046 |
| Valine, leucine and isoleucine degradation | 40 | 1.57 | 5 | 0.0182 | 1 | 0.0844 |
| Nitrogen metabolism | 6 | 0.236 | 2 | 0.0206 | 1 | 0.0905 |
| Starch and sucrose metabolism | 18 | 0.708 | 3 | 0.031 | 1 | 0.129 |
| Cysteine and methionine metabolism | 33 | 1.3 | 4 | 0.0377 | 1 | 0.149 |
| Pentose phosphate pathway | 22 | 0.866 | 3 | 0.0524 | 1 | 0.197 |
| Galactose metabolism | 27 | 1.06 | 3 | 0.0867 | 1 | 0.311 |
| Inositol phosphate metabolism | 30 | 1.18 | 3 | 0.111 | 1 | 0.381 |
| Histidine metabolism | 16 | 0.63 | 2 | 0.128 | 1 | 0.406 |
| Glycerolipid metabolism | 16 | 0.63 | 2 | 0.128 | 1 | 0.406 |
| Pentose and glucuronate interconversions | 18 | 0.708 | 2 | 0.156 | 1 | 0.473 |
| Glycerophospholipid metabolism | 36 | 1.42 | 3 | 0.166 | 1 | 0.485 |
| Amino sugar and nucleotide sugar metabolism | 37 | 1.46 | 3 | 0.176 | 1 | 0.491 |
| Linoleic acid metabolism | 5 | 0.197 | 1 | 0.182 | 1 | 0.491 |
| Pyrimidine metabolism | 39 | 1.53 | 3 | 0.196 | 1 | 0.491 |
| beta-Alanine metabolism | 21 | 0.826 | 2 | 0.199 | 1 | 0.491 |
| Sphingolipid metabolism | 21 | 0.826 | 2 | 0.199 | 1 | 0.491 |
| Steroid biosynthesis | 42 | 1.65 | 3 | 0.227 | 1 | 0.53 |
| Propanoate metabolism | 23 | 0.905 | 2 | 0.228 | 1 | 0.53 |
| Thiamine metabolism | 7 | 0.275 | 1 | 0.245 | 1 | 0.554 |
| Lysine degradation | 25 | 0.984 | 2 | 0.258 | 1 | 0.566 |
| Ascorbate and aldarate metabolism | 8 | 0.315 | 1 | 0.275 | 1 | 0.569 |
| Taurine and hypotaurine metabolism | 8 | 0.315 | 1 | 0.275 | 1 | 0.569 |
| Fatty acid biosynthesis | 47 | 1.85 | 3 | 0.281 | 1 | 0.569 |
| Ubiquinone and other terpenoid-quinone biosynthesis | 9 | 0.354 | 1 | 0.304 | 1 | 0.6 |
| Biotin metabolism | 10 | 0.393 | 1 | 0.331 | 1 | 0.625 |
| Porphyrin and chlorophyll metabolism | 30 | 1.18 | 2 | 0.332 | 1 | 0.625 |
| alpha-Linolenic acid metabolism | 13 | 0.511 | 1 | 0.408 | 1 | 0.749 |
| Nicotinate and nicotinamide metabolism | 15 | 0.59 | 1 | 0.454 | 1 | 0.815 |
| Tyrosine metabolism | 42 | 1.65 | 2 | 0.499 | 1 | 0.876 |
| Primary bile acid biosynthesis | 46 | 1.81 | 2 | 0.548 | 1 | 0.912 |
| Fructose and mannose metabolism | 20 | 0.787 | 1 | 0.554 | 1 | 0.912 |
| Selenocompound metabolism | 20 | 0.787 | 1 | 0.554 | 1 | 0.912 |
| Purine metabolism | 65 | 2.56 | 2 | 0.737 | 1 | 1 |
| Arachidonic acid metabolism | 36 | 1.42 | 1 | 0.768 | 1 | 1 |
| Fatty acid elongation | 38 | 1.5 | 1 | 0.787 | 1 | 1 |
| Fatty acid degradation | 39 | 1.53 | 1 | 0.795 | 1 | 1 |
| Tryptophan metabolism | 41 | 1.61 | 1 | 0.811 | 1 | 1 |
| Steroid hormone biosynthesis | 85 | 3.34 | 1 | 0.97 | 1 | 1 |
